# Self-reports vs clinician ratings of efficacies of psychotherapies for depression: a meta-analysis of randomized trials

**DOI:** 10.1017/S2045796025000095

**Published:** 2025-03-06

**Authors:** Clara Miguel, Mathias Harrer, Eirini Karyotaki, Constantin Yves Plessen, Marketa Ciharova, Toshi A. Furukawa, Ioana A. Cristea, Pim Cuijpers

**Affiliations:** 1 Department of Clinical, Neuro and Developmental Psychology, Amsterdam Public Health Research Institute, Vrije Universiteit Amsterdam, Amsterdam, Netherlands; 2 Psychology & Digital Mental Health Care, Technical University Munich, Munchen, Germany; 3 Clinical Psychology & Psychotherapy, Friedrich-Alexander University Erlangen-Nuremberg, Erlangen, Germany; 4 WHO Collaborating Centre for Research and Dissemination of Psychological Interventions, Vrije Universiteit Amsterdam, Amsterdam, Netherlands; 5 Department of Psychosomatic Medicine, Charité – Universitätsmedizin Berlin, corporate member of Freie Universität Berlin and Humboldt-Universität zu Berlin, Berlin, Germany; 6 Department of Health Promotion and Human Behavior, Kyoto University Graduate School of Medicine/School of Public Health, Kyoto, Japan; 7 Department of General Psychology, University of Padova, Padova, Italy

**Keywords:** blinding, clinician-rated scales, depression measurement, self-reports, psychotherapy

## Abstract

**Aims:**

The comparability between self-reports and clinician-rated scales for measuring depression following treatment has been a long-standing debate, with studies finding mixed results. While the use of self-reports in psychotherapy trials is very common, it has been widely assumed that these tools pose a validity threat when masking of participants is not possible. We conducted a meta-analysis across randomized controlled trials (RCTs) of psychotherapy for depression to examine if treatment effect estimates obtained via self-reports differ from clinician-rated outcomes.

**Methods:**

We identified studies from a living database of psychotherapies for depression (updated to 1 January 2023). We included RCTs measuring depression at post-treatment with both a self-report and a clinician-rated scale. As our main model, we ran a multilevel hierarchical meta-analysis, resulting in a pooled differential effect size (Δ*g*) between self-reports and clinician ratings. Moderators of this difference were explored through multimodel inference analyses.

**Results:**

A total of 91 trials (283 effect sizes) were included. In our main model, we found that self-reports produced smaller effect size estimates compared to clinician-rated instruments (Δ*g* *=* 0.12; 95% CI: 0.03–0.21). This difference was very similar when only including trials with masked clinicians (Δ*g* *=* 0.10; 95% CI: 0.00–0.20). However, it was more pronounced for unmasked clinical ratings (Δ*g* *=* 0.20; 95% CI: −0.03 to 0.43) and when trials targeted specific population groups (e.g., perinatal depression) (Δ*g* *=* 0.20; 95% CI: 0.08–0.32). Effect sizes between self-reports and clinicians were identical in trials targeting general adults (Δ*g* *=* 0.00; 95% CI: −0.14 to 0.14).

**Conclusions:**

Self-report instruments did not overestimate the effects of psychotherapy for depression and were generally more conservative than clinician assessments. Patients’ perception of improvement should not be considered less valid by default, despite the inherent challenge of masking in psychotherapy.

## Introduction

Psychotherapy is one of the first-line approaches for the treatment of depression (World Health Organization, 2016). The effects of psychological interventions for depression have been well established in more than 800 randomized trials, most of them conducted over the last 10 years (Cuijpers *et al.*, 2022). In most psychotherapy trials, the success of interventions is examined through scales measuring depression symptoms before and after the treatment, which can be divided into two major categories: clinician-rated versus patient-rated or self-report instruments (Möller, 2000).

Traditionally, clinician-rated scales have been assumed to provide a more objective and standardized measurement of patients’ symptoms (Möller, 2000), being considered the ‘gold standard’ (e.g., the Hamilton Rating Scale for Depression (HRSD; Hamilton, 1960)). However, their administration requires time investment, trained personnel, and holds the possibility of clinician biases (e.g., over-confidence, unmasked assessment, etc.) (Lewis, 1991). In the psychotherapy field, depression is commonly assessed using self-report instruments, such as the Beck Depression Inventory (BDI-II; Beck *et al.*, 1996). Self-report instruments provide a more subjective and patient-focused measurement of depression and have the advantage of highly reducing time investment and costs.

Whether self-report instruments and clinician-rated scales yield comparable results for measuring depression has been a long-standing debate, with primary studies finding mixed results. For example, an earlier study by Rush and colleagues (2006) showed little differences in predicting response and remission when administering the same instruments through self-report and clinician-rated versions. Similarly, Zimmerman *et al.* (2018) found a high level of agreement between widely used clinician ratings and self-reports in classifying treatment responders. On the other hand, Uher and colleagues (2012) observed that each of these types of ratings provide unique information for assessing pharmacotherapies’ efficacy and suggested that self-reports should be preferred if only one measure could be used. In the same vein, a recent study in routine inpatient settings found little agreement between clinician assessments and patient-reported outcomes on detecting failure to achieve a clinically significant change, and no agreement on detecting deterioration (Kaiser *et al.*, 2022).

While most of the evidence focuses on antidepressant treatment, the implications of this question are of particular relevance for the psychotherapy field. In psychotherapy trials, masking participants is typically unfeasible, or even impossible (Baskin *et al.*, 2003; Munder and Barth, 2018). Consequently, self-reports rely on unmasked participants to self-assess their symptoms after receiving the treatment or the control condition. Being aware of treatment allocation may lead to two types of bias, as those who know that they are on a control or ineffective intervention may seek additional treatments, which can undermine the comparisons. A more subtle form of performance bias is the influence of expectation (when patients know they are allocated to an active treatment) or disappointment (when patients know they have allocated to the control condition). These factors may compromise the internal validity of the trial but can be said to reflect true changes in the patient’s status. Another form of bias introduced by unmasking is assessment bias, where individuals evaluating a condition in which they have conscious or unconscious vested interests may unintentionally influence their assessment. This type of bias is different from performance bias because this does not reflect the true status of the patients (Boutron *et al.*, 2019). Therefore, it is essential to understand if treatment effect estimates obtained via self-reports systematically differ from (masked) clinician-rated outcomes.

To the best of our knowledge, only one study has systematically examined this question across psychotherapy trials for depression (Cuijpers *et al.*, 2010). This earlier meta-analysis suggested that clinician-rated scales might result in larger effect estimates than self-reports. However, it included studies published up to 2009, while there has been a stark increase of trials after 2010 (Cuijpers *et al.*, 2023). Including a larger sample of trials in an up-to-date study would allow us to apply state-of-the-art meta-analytic techniques for dealing with multiple outcomes within studies, such as multilevel meta-analysis, obtaining a more robust understanding of this question.

Therefore, we conducted a meta-analysis to examine systematic differences in effect estimates between self-reports and clinician assessments across randomized trials of psychotherapy for depression.

## Methods

### Identification and selection of studies

The protocol and analysis plan of the current study was registered in OSF (https://osf.io/c9tbz). The trials included in this study are part of a living meta-analytic database on psychological treatments of depression (Cuijpers *et al.*, 2023) (www.metapsy.org; doi:10.17605/OSF.IO/825C6). For developing this database, we periodically search four major bibliographical databases (PubMed, PsycINFO, Embase and Cochrane Library). Two independent researchers screened all titles, abstracts and full texts. All the procedures and documentation (e.g., search strings) are available at www.metapsy.org.

We used the latest version of the database, up to 1 January 2023. We included RCTs comparing (1) psychological interventions (2) against control conditions (waiting-list, care-as-usual, other control condition such as attention placebo) in (3) adults with a diagnosis or elevated symptoms of depression. We included trials that (4) measured depression at post-treatment with at least one self-report (e.g., BDI) and one clinician-rated (e.g., HRDS) scale. Examining trials that include both self-reports and clinician-rated instruments will allow us to more easily rule out potential confounding in our analysis of this difference of interest.

We excluded studies in which it was explicitly stated that a commonly used self-report measure was administered by an interviewer (e.g., reading the questions to the participants through a telephone interview). Any type of psychological treatment (cognitive-behaviour therapy [CBT], ‘third wave’ therapies, etc.) delivered as individual, group or guided self-help was included.

### Data extraction and risk of bias (RoB) assessment

As part of the broader meta-analytic database, we extracted participant characteristics, characteristics of the psychological interventions and general characteristics of the studies. The details of these characteristics can be found at the website of the project (docs.metapsy.org/databases/depression-psyctr/) and in the Supplement (eResults).

We assessed the RoB using four domains of the Cochrane’s Risk of bias revised tool (RoB 2) (Sterne *et al.*, 2019), evaluating biases in RCTs arising from the randomization process (domain 1), deviations from the intended interventions (domain 2), missing outcome data (domain 3) and the selection of the reported result (domain 5). A score of low risk, some concerns or high RoB was assigned to each of these four domains based on responses to a series of signalling questions. An overall RoB score was obtained for each study. A study was rated as overall low RoB when it scored low risk in all four domains. High risk was rated when one domain had a high-risk score or when multiple domains (i.e., more than three of the four domains) had a some concerns score. Finally, a trial was rated as some concerns when one or two domains had a some concerns score. This overall RoB score was used in the moderation analyses.

The RoB 2 tool also assesses biases arising from the measurement of the outcome (domain 4), with the central question of whether the assessment of the outcome is likely to be influenced by the knowledge of treatment allocation. This is the primary analysis examined in this study. Therefore, we collected detailed information about this domain, but we did not include it in the overall RoB score used in the moderator analysis to avoid an overlap between the independent and the dependent variable. We considered clinician-rated scales as masked when it was explicitly stated that outcome assessors were masked to treatment allocation. When no information was provided in the trial report, we conservatively assumed them as unmasked.

Data extraction and RoB assessments were conducted by two independent researchers.

## Outcome measures

We included any validated self-report and clinician-rated scale for depression, for which we calculated an effect size estimating the difference between the psychotherapy and the control groups at post-treatment.

Hedges’ *g* [small sample bias corrected standardized mean difference (SMD)] and the associated sampling variance were calculated for each self-report and clinician-rated measure within a trial. If studies assessed depressive symptom severity using more than one instrument, effect sizes were calculated for all eligible instruments. For example, when two clinician-rated instruments and one self-report instrument were reported within a study, we calculated effect sizes for the three of them and included all three measures in the analyses. We calculated effect sizes based on means and standard deviations at post-test, but if these were not reported, we used other available data such as change scores or binary outcomes.

## Meta-analyses

In the primary analysis, our outcome of interest was the difference in effect estimates (SMDs) using self-reports and those using observer-ratings of the same intervention-control comparison. One trial may administer two or more self-report scales and two or more observer-rating scales and may include multiple comparisons (e.g., two active arms and one control arm). To account for this multi-level clustering within studies, we pooled effect sizes using a bivariate four-level hierarchical meta-analysis model, with separate sampling variance-covariance matrices constructed for each study. These matrices accounted for dependencies due to (1) multiple measurements of the same outcome (clinician-rated or self-reported depression) using different instruments [e.g., BDI, Center for Epidemiologic Studies Depression Scale (CES-D); assuming *ρ* = 0.9]; (2) assumed correlations between self- and clinician-rated depression, which were derived from previous estimates in the literature (*ρ* = 0.8; Bukumiric *et al.*, 2016; Uher *et al.*, 2012) and (3) dependencies introduced by the inclusion of trials with multiple arms (in which we derived the correlation from the sample size of each trial arm (Borenstein *et al.*, 2011)). We assumed a doubly nested random effects structure (effects *in* [clinician, self-report] outcomes *in* studies), which means that three heterogeneity variance components were estimated. Our main analysis included a fixed stratification term for rating type (self-report, clinician). This allows to compare the overall clinician-rated and self-report effect size across studies that report both, while accounting for complex effect size dependencies. The differential effect size resulting from comparing the effects of self-reports with clinician ratings estimated from these models was indicated with Δ*g*. A positive Δ*g* value indicates larger effects for clinician ratings. We interpreted the clinical relevance of an effect difference using the threshold of *g* = 0.24, based on previous research on depression (Cuijpers *et al.*, 2014). To test the robustness of our primary analysis, we performed several sensitivity analyses using different methods for pooling (eMethods). Exact formulas for all employed models are provided in the Supplement.

We also explored if there were study characteristics that predicted the degree to which patient and clinician-rated outcomes differed in a study by employing meta-analytic multimodel inference (Anderson, 2007; Buckland *et al.*, 1997). We specified the following potential moderators of the effect size difference Δ*g* between patient and clinician-reported outcomes: masking of the assessor (masked vs unmasked, considering self-reports as unmasked), overall RoB score (high/some concerns vs low risk), target group (specific subgroups vs general adults), control group (waitlist vs other controls), country (western vs non-western) and type of treatment (CBT vs other). The next step involved fitting a separate (meta-regression) model for each possible combination of these predictors. This means that the effect of each predictor is estimated in many multivariable models that control for the effect of other predictors, including their interaction. Based on the fit of each model (measured by the corrected Akaike information criterion), it is possible to create a weighted average for each variable, representing its importance in predicting effect size differences across all fitted models. For computational reasons, we restricted models to a maximum of 6 terms, leading to a total of 56,734 fitted models.

Finally, to explore the impact of important trial characteristics in our primary analysis we repeated our main model but (1) stratified by masking of clinicians, (2) stratified by the population group (general adults vs specific groups), (3) excluded effect sizes based on the Geriatric Depression Scale (GDS) and Edinburgh Postnatal Depression Scale (EPDS) (focused on geriatric and perinatal depression) and (4) only analysed trials reporting both the BDI (I, II) and the HRSD-17 (and related versions) while excluding all other instruments.

To guard against potential model misspecification, all statistical tests and confidence intervals were obtained using the CR2 cluster-robust variance estimator (Pustejovsky and Tipton, 2018). The certainty of the evidence was evaluated using GRADE (Guyatt *et al.*, 2011) (more details about the ratings can be found in eResults). Analyses were conducted using R (version 4.2.0) with the packages *meta, metapsyTools, metafor, emmeans* and *metaSEM*.

## Results

### Study inclusion and characteristics

The PRISMA flowchart describing the inclusion process and the references of included studies are available in the Supplement (eResults). A total of 91 RCTs met inclusion criteria, with 128 comparisons between psychotherapy and control conditions (due to trials with multiple arms), including 7250 participants. A narrative description of the most relevant study characteristics and RoB is presented in the Supplement (eResults), together with a table providing this information for each included study.

Seventeen different self-report and clinician-rated instruments were administered, resulting in a total of *k* = 283 post-treatment effect sizes (37 trials included 3 or more instruments) (Table 1). The HRSD-17 and its related versions (e.g., HRSD-24) were the most used clinician-rated scale (*k* = 116), and the BDI (I and II) were the most included self-reports (*k* = 91). The exact scales used in each trial are presented in the Supplement (Table S1).

**Table 1.** Aggregated overview of the instruments used in the 91 trials, contributing to a total of *k* = 283 effect sizes across studies

Rating	Instrument	*k*
Clinician-rated scales	HRSD*	116
	MADRS-cr	8
	IDS	5
	QIDS-cr	2
Self-report scales	BDI-I	61
	BDI-II	30
	GDS	14
	PHQ-9	13
	EPDS	8
	CES-D	5
	SCL-90	5
	QIDS-sr	4
	MMPI-D	4
	HADS-D	3
	IDAS-D	2
	SDS	1
	PROMIS	1
	BASIS-24-D	1

*Notes:*

Full names of the instruments and their references are available in the Supplement (eResults).

*k*: number of effect sizes.

* Includes HRSD-17 and all other variants (e.g., HRSD-24).

Most of the trials (*n* = 74; 81%) specified that the personnel administering the clinician-rated scales at post-treatment were masked to treatment allocation. The remaining 17 trials (19%) were considered in our analyses as not masked, either not reporting information about masking (*n* = 13) or explicitly stating that assessors were not masked (*n* = 4).

### Differences between self-reports and clinician-rated scales in all included studies

**Figure 1.**
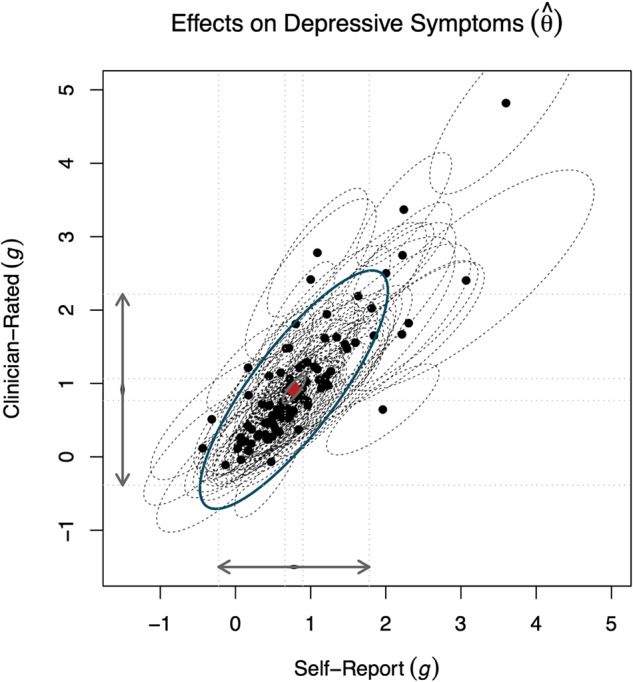
*XY* plot of the estimated bivariate effects for self-reports and clinician-rated scales.

*Notes.* This plot represents the estimated bivariate effects for self-reports (*X*-axis) and clinician-rated outcomes (*Y*-axis). Black dots represent the effect sizes in individual studies, with their 95% confidence intervals shown as an ellipsis with a dotted line. The red diamond at the centre shows the pooled effect size, with the 95% confidence interval in a grey ellipsis and the prediction interval in a blue ellipsis.

Figure 1 shows the estimated bivariate effects for self-reports and clinician-rated outcomes, and the results of our main meta-analytical model are presented in Table 2. The pooled effect was *g* = 0.75 (95% CI: 0.63–0.87) for self-report and *g* = 0.87 (95% CI: 0.72–1.01) for clinician-rated outcomes. The overall differential effect size between rating types was Δ*g* *=* 0.12 (95% CI: 0.03–0.21; *p* = 0.01). There was substantial between-study heterogeneity, with *I*^2^ = 58% and *τ*^2^ = 0.25 (eResults, Table S3), and prediction intervals were very wide and crossed 0 in all analyses. The certainty of the evidence was rated as low, according to GRADE (eResults). Sensitivity analyses using different methods for pooling showed a similar pattern in effect sizes as our main model (Table S2).

**Table 2.** Pooled effects and contrasts between self-reports and blinded and unblinded clinician ratings

Main model	*k*	*g*^a^	95% CI	PI	*p*_contrasts_
*Pooled effects*					
Self-reports	152	0.747	0.63 to 0.87	−0.40 to 1.90	-
Clinician-rated	131	0.866	0.72 to 1.01	−0.29 to 2.02	-
*Contrast (Δg)*					
Self-reports vs clinician-rated	283	0.119	0.03 to 0.21	−1.04 to 1.27	0.01

*Notes:*

a *g* refers to the pooled Hedges’ *g* in the section of pooled effects, and the differential Hedges’ *g* (Δ*g*) for the section of contrasts. *Δg* *=* Differential effects between self-reports and clinician-rated instruments. A positive value indicates larger effects for clinician-rated instruments.

95% CI: 95% confidence interval; PI: prediction interval; *k* = total number of effect sizes included in the analysis.

### Moderators of the difference between self-reports and clinician ratings

**Figure 2.**
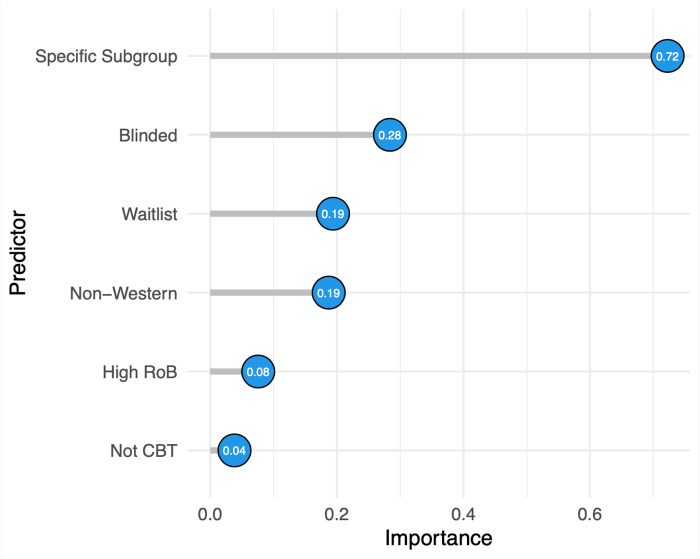
Importance values of moderators of the effect contrasts between self-reports and clinician-rated outcomes examined in the multimodel inference analysis.

*Notes*. Importance values ranging from 0 to 1. These values are based on the corrected Akaike information criterion (AICc) of each model, indicating how well the model fits the data and how much weight that specific model has in the overall multimodel inference analysis.

Multimodel inference was used to explore moderators of the differences between patient- and clinician-rated outcomes investigated in our main model while controlling for the influence of other predictors. As can be seen in Figure 2, the most important predictors were whether the study had been conducted with a specific population group (e.g., older adults, patients with somatic disorders, women with perinatal depression), with an importance value of 0.70 out of a maximum of 1, and the masking of clinicians, with 0.28. The remaining variables were use of waitlist controls (0.19), non-western countries (0.19), overall RoB score of the other domains (0.08) and type of therapy (non-CBT) (0.04). Further details are reported in the Supplement.

### Sensitivity analyses on important trial characteristics

In our multimodel inference analysis, specific subgroup and blinding emerged as the two most important predictors of effect difference between clinician ratings and self-reports. Therefore, we explored the impact of these trial characteristics in our primary analysis (Table 3). First, when stratifying by masking of assessors, unmasked clinician ratings resulted in larger effects than self-reports, with a differential effect of Δ*g* *=* 0.20 (95% CI: −0.03 to 0.43) (certainty according to GRADE: Very low). Masked clinician ratings and self-reports had a smaller difference of Δ*g* *=* 0.10 (95% CI: 0.00–0.20) (certainty according to GRADE: Low). Second, when we stratified by the population group, self-reports and clinical ratings resulted in the same estimates for the group of general adults (Δ*g* = 0.001, 95% CI: −0.14 to 0.14), while there was a difference of Δ*g* = 0.20 (95% CI: 0.08–0.32) for the specific population groups. Similarly, excluding effect sizes based on the GDS and EPDS resulted in little differences between self-reports and clinician ratings (Δ*g* = 0.05; 95% CI: −0.03 to 0.43). Finally, when only analysing trials that reported both the BDI and HRSD, the outcomes between the two ratings were again comparable (Δ*g* = 0.04; 95% CI: −0.07 to 0.15).

**Table 3.** Sensitivity analyses on important trial characteristics

Contrasts	*k*	Δ*g*	95% CI	PI	*p_contrasts_*
Blinding of assessors					
Self-reports vs unmasked clinicians	49	0.20	−0.03 to 0.43	−1.07 to 1.47	0.088
Self-reports vs masked clinicians	234	0.10	0.00 to 0.20	−1.04 to 1.24	0.044
Target population					
General adults	120	0.00	−0.14 to 0.14	−1.17 to 1.17	0.991
Specific population groups^a^	163	0.20	0.08 to 0.32	−0.98 to 1.39	0.001
Instruments					
Excluding GDS and EPDS	248	0.05	−0.03 to 0.12	−0.98 to 1.08	0.250
Only trials reporting BDI and HRSD	162	0.04	−0.07 to 0.15	−1.01 to 1.09	0.515

*Notes:*

95% CI: 95% confidence interval; PI: prediction interval; *k* = number of effect sizes included in the analysis.

Δ*g* = Differential effects between self-reports and clinician-rated instruments. A positive value indicates larger effects for clinician-rated instruments.

GDS: Geriatric Depression Scale; EPDS: Edinburgh Postnatal Depression Scale.

BDI (Beck Depression Inventory) includes version I and II; HRSD (Hamilton Rating Scale for Depression) includes all related versions (17-, 24-item, etc.). This analysis includes only trials that report both groups of instruments.

a Specific population groups included older adults, women with perinatal depression, patients with depression comorbid to physical illnesses and other heterogeneous small subgroups (e.g., veterans, depression co-occurring with autism, impoverished mothers).

## Discussion

This meta-analysis examined whether clinician-rated and self-report depression scales lead to differential outcomes in psychotherapy trials. We included 91 RCTs that measured depression at post-treatment with at least a self-report and a clinician-rated scale, resulting in a total of 283 effect sizes. Overall, we found that self-reports had somewhat smaller post-treatment effect sizes compared to clinician-rated instruments, with a differential effect size of Δ*g* = 0.12 (95% CI: 0.03–0.21). Through multimodel inference analysis, we observed that the most important predictors were whether the trial was focused on specific population groups (e.g., perinatal depression, older adults) and the masking of clinicians. When taking the masking into account, the difference between masked clinician assessments and self-reports was slightly smaller (Δ*g* = 0.10, 95% CI: 0.00–0.20). However, when assessors were not masked (or masking was unclear), this difference was larger, with Δ*g* = 0.20 (95% CI: −0.03 to 0.43). Similarly, when the target sample was a specific population (with population-specific self-reports, e.g., EPDS, GDS), clinician ratings resulted in larger effect sizes than self-reports, with Δ*g* = 0.20 (95% CI: 0.08–0.32). These differences are probably clinically meaningful. Nevertheless, when we excluded specific populations and population-specific scales, the outcomes of self-report and clinician-rated scales were practically identical (Δ*g* = 0.00–0.05).

These findings have implications for research synthesis in the field of psychotherapy for depression. First, when examining the field as a whole, aiming at a comprehensive synthesis of all types of studies, populations and assessment instruments, self-reports and (masked) clinician ratings do not seem to exhibit clinically important differences. Unmasked assessors’ ratings appeared to overestimate treatment effects compared to self-reports, with a probable clinically meaningful difference. There was a small number of trials with unmasked clinicians (*n* = 17), thus this specific analysis should be interpreted more carefully. Second, when aiming at a more focused synthesis on specific population groups, often using population-specific instruments, a more detailed assessment of systematic differences between self-reports and clinician ratings would be warranted. Based on our results, there is a possibility that in these studies self-reports underestimate the outcomes or clinician-rated scales overestimate them. The underlying reasons for the differences between the ratings of population specific trials and those aimed at general population remain unclear. It is uncertain whether this difference is attributable to the specific instruments used in these trials or to other characteristics of trials or the population under study. Some studies have suggested patient-level differences in the disagreements between self-reports and clinician ratings (e.g., female gender, higher levels of anxiety, younger age, chronicity of depression) (Carter *et al.*, 2010; Dunlop *et al.*, 2011; Hershenberg *et al.*, 2020; Rane *et al.*, 2010).

The comparability of our results with previous studies is difficult. Previous studies had very different designs (Kaiser *et al.*, 2022), were mostly conducted in the context of drug trials (e.g., Dunlop *et al.*, 2010; Uher *et al.*, 2012; Zimmerman *et al.*, 2018) or were broad meta-epidemiological studies examining blinding across all medical specialties, classifying interviewer and patient-rated scales in the same level (subjective), but not differentiating between them (e.g., Wang *et al.*, 2024; Moustgaard *et al.*, 2020; Savovic *et al.*, 2018). Examining these two ratings separately is crucial for answering this pragmatically important question in mental health and psychotherapy research. Only one previous study comprehensively examined this question in psychotherapy trials (Cuijpers *et al.*, 2010). The results of this earlier meta-analysis suggested that clinician ratings yielded larger effects than self-reports, with a difference that could be clinically meaningful (Δ*g* = 0.20). Our results replicate the main implication from this previous study, indicating that self-reports are not associated with an overestimation of psychotherapy effects. However, we found that the difference between self-reports and clinician ratings is smaller (Δ*g* = 0.12), and even smaller in trials recruiting general adult samples or when compared to masked observer ratings. One reason for this difference could be that newer studies tend to more frequently mask their assessors. Since the publication of the updated CONSORT guidelines in 2010 (Schulz *et al.,* 2010) and the Cochrane RoB tool in 2011 (Higgins *et al.,* 2011), the overall reporting and methodology of clinical trials have significantly improved, including the adoption of more rigorous reporting of blinding procedures. The current study includes 43 recent RCTs not analysed in Cuijpers *et al.* (2010), nearly doubling the sample size. This larger sample size has allowed us to apply more sophisticated methods than those used in Cuijpers *et al.* (2010), such as multilevel meta-analytic techniques for dealing with multiple outcomes within studies. This improved analytic approach, applied to a large dataset of 91 RCTs with 7250 participants, offers the most robust and reliable answer to this research question in our field.

These results can be interpreted in the context of two longstanding debates. The first debate involves the choice of outcome measures for assessing depression severity following treatment. Some authors suggest that best practice would be to include both clinician and self-reported scales (e.g., Uher *et al.*, 2012), while others proposed that these could be used interchangeably to save costs (e.g., Rush *et al.*, 2006). Current depression scales are very different from each other, regardless of who the assessor is, and probably measure different aspects of depression (Fried *et al.*, 2022). The choice of outcome measures should be made taking the specific context into account. For example, in low-resourced settings, the use of self-reports could offer advantages for implementation, facilitate participant recruitment and increase the generalizability of the findings.

The second debate involves the use of self-reports and their potential RoB when evaluating the effects of psychological interventions. One important source of bias in clinical trials entails the conscious (e.g., interests) and unconscious predispositions (e.g., hope) in the assessors performing the ratings. In the absence of participant masking in psychotherapy trials, all self-report instruments are inherently unmasked and, thus, at risk for these (unconscious) predispositions from the participants. Our study provides additional evidence for the application of RoB assessments in the field of psychotherapy. We found that it is very unlikely that participants overestimate their assessments after receiving psychotherapy compared to masked clinician ratings. Therefore, self-reports might not necessarily pose a default RoB in psychotherapy trials for depression.

We observed an overall small difference of 0.12 between self-reports and clinician ratings (0.10 when clinicians are masked), with self-reports producing more conservative effect sizes. Based on these findings, one could argue that clinician-ratings either overestimate the true treatment effect, or that self-reports underestimate it. Which of the two applies is difficult to ascertain, since this presumes that the ‘true’ effect of a treatment can be quantified without measurement error. Depression is a highly heterogeneous condition, and it is also possible that self-report and observer-rated instruments simply measure different aspects of the construct ‘depression’ (Borsboom, 2017; Fried, 2017). We can infer that some overestimation exists when unmasked clinicians are used, likely because such ratings are more susceptible to conscious and unconscious biases of the observer. In the case of masked clinicians, reasons for the effect difference compared to self-reports are less clear. It could be that clinicians are more sensitive to change in comparison to patients, by observing, e.g., changes in behaviour or facial expressions, while patients are more pessimistic in their self-evaluations. It could also be that patients have more information about their symptoms and can estimate more realistically whether a treatment was effective. What we can conclude is that patient ratings do not seem to produce systematically higher treatment estimates than blinded observer ratings, as one could assume given the fact that patients are, by default, unblinded to their treatment condition.

Our results support the notion that patients’ perception of improvement should not be considered less valid because of the impossibility of masking. Lack of masking in psychotherapy might also be a necessary working mechanism of interventions that should not be directly disqualified. Some theoretical models of psychotherapy stress that ‘contextual factors’ such as treatment expectations are an essential working factor of treatments (Doering *et al.*, 2018; Wampold and Imel, 2015). Thus, being aware of receiving a ‘bona fide’ psychological treatment might be a precondition to its effectiveness.

Our findings should be interpreted considering some limitations. First, different instruments were pooled in two major categories that were compared with each other, while these different instruments might be measuring different aspects of symptom improvement (Fried *et al.*, 2022), or might be of very different nature due to being addressed at a specific population group. Different confounders such as the quality and psychometric properties of the instruments could also influence our findings. Second, there is still some uncertainty regarding the relationship between self-reports and clinician ratings, as can be seen by the wide prediction intervals in all the analyses. This might not be necessarily due to the instability of such relationship, as it could also be due to the previously mentioned problems in depression measurement. Another limitation is the applicability of the estimate we used as a clinically meaningful threshold in the context of our study (Cuijpers *et al.*, 2014). This is a rough indication, and it is possible that for a comparison between two techniques for measuring depression the threshold is actually smaller. Moreover, the results of our study should be interpreted considering its observational nature, in which trial and participant characteristics could be confounding the analyses. However, a strength of our study is that we only included trials administering both types of ratings, which facilitates that confounding can be more easily ruled out for this particular difference of interest.

## Conclusion

The results from our synthesis of 91 randomized trials on psychotherapy for depression showed that self-reports produced somewhat smaller effect sizes compared to clinician-rated instruments, with an overall difference in effects of Δ*g* = 0.12. There were larger differential outcomes between self-reports and clinician ratings in trials targeting specific population groups, while effect sizes from these ratings were identical in trials targeting general adults. In the context of psychotherapy for depression, self-reports are not associated with an overestimation of treatment effects.

## Supporting information

Miguel et al. supplementary material Miguel et al. supplementary material

## Data Availability

Data and analysis code are available at the Open Science Framework project (https://osf.io/jfwkr/). The broader meta-analytic database from which the included studies were selected is also available at .

## Supplementary material

The supplementary material for this article can be found at https://doi.org/10.1017/S2045796025000095.
