# Supplementary material for: Self-reports vs clinician ratings of efficacies of psychotherapies for depression: a meta-analysis of randomized trials
Source: Epidemiol Psychiatr Sci. 2025 Mar 6;34:e15. doi: 10.1017/S2045796025000095 (PMC11886967; doi:10.1017/S2045796025000095)
Supplement: Miguel et al. supplementary material [file S2045796025000095sup001.docx]

**Supplementary material**

**Table of Contents**

[eMethods 2](#_Toc187248056)

[Amendments to protocol 2](#_Toc187248057)

[Model formulas and sensitivity analyses on methodological decisions 3](#_Toc187248058)

[eResults 4](#_Toc187248059)

[Figure S1. PRISMA Flowchart 4](#_Toc187248060)

[References of the included studies 5](#_Toc187248061)

[Characteristics of the included studies 12](#_Toc187248062)

[Table S1. Characteristics of the 91 included studies 13](#_Toc187248063)

[List of instruments used in the included studies 18](#_Toc187248064)

[Table S2. Sensitivity analysis on methodological decisions 19](#_Toc187248065)

[Table S3. Heterogeneity in the models (I^2^ and tau^2^) 20](#_Toc187248066)

[Multimodel inference: best models 21](#_Toc187248067)

[References of the instruments 22](#_Toc187248068)

[GRADE assessments 23](#_Toc187248069)

## eMethods

### Amendments to protocol

We conducted two post-hoc sensitivity analyses that were not registered, which were carried out to further explore the robustness of our results. One analysis aims at repeating the main model but lowering the levels of assumed correlations in the variance-covariance matrices. The second exploratory sensitivity analysis aimed at examining the impact of specific population groups on our results, given that this variable emerged as the most important predictor in the multimodel inference analysis.

### Model formulas and sensitivity analyses on methodological decisions

Our main pooling method (“main model”) was a four-level hierarchical meta-analysis model. For this model, we assumed a doubly nested random effects structure (effects *in* [clinician, self-report] outcomes *in* studies), which means that three heterogeneity variance components are estimated across these levels: study, rating (self-report vs. clinician), and outcome or instrument level (effect size for a specific instrument). A generalized formula for this model can be denoted like so:

$$y_{ijk}=\mathbf{x}_{ijk}\boldsymbol{\beta}+u_{k}^{\left( 1 \right)}+u_{jk}^{\left( 2 \right)}+u_{ijk}^{\left( 3 \right)}+e_{ijk}$$

Where $y_{ijk}$ is the calculated effect size estimate $i$ in rating cluster $j$ included in study $k$, $\boldsymbol{\beta}$ is a generic vector of $p$ regression coefficients, and $\mathbf{x}_{ijk}$ a row vector of $p$ covariates additionally entered into the model. The error terms $u$ represent the (nested) random effects, with $\mathrm{Var}\left[ u_{k}^{\left( 1 \right)} \right]=\tau_{(1)}^{2}$ being the between-study heterogeneity variance as typically calculated in random-effect meta-analyses. As described in the methods section, the error terms $e_{ijk}$ were assumed to be correlated. Thus, a variance-covariance matrix $\mathbf{V}_{k}$ was constructed for each model, using the (assumed to be known) sampling variances $s_{ij}^{2}$ of all included effects:

$$\mathbf{V}=\left[ \begin{matrix} \mathbf{V}_{1} & \begin{matrix} & \end{matrix} \\ \begin{matrix} \\ \end{matrix} & \begin{matrix} \begin{matrix} \ddots& \end{matrix} \\ \begin{matrix} & \mathbf{V}_{K} \end{matrix} \end{matrix} \end{matrix} \right].$$

Next to our primary analysis, we performed two planned sensitivity analyses with different methods for pooling the effect sizes (i.e., different model specifications). First, we pooled effects after pre-aggregating them on an outcome level (clinician vs. self-report) using the approximate variance-covariances constructed for the primary analysis. This pre-aggregation avoids the need to estimate a complex nesting structure and means that a simpler bivariate meta-analysis model with correlated random effects can be employed. This approach also avoids modeling rating clusters as random. The formula for the model thus simplifies to:

$$\left[ \begin{matrix} y_{k}^{\mathrm{sr}} \\ y_{k}^{\mathrm{cr}} \end{matrix} \right]=\left[ \begin{matrix} \mu^{\mathrm{sr}} \\ \mu^{\mathrm{cr}} \end{matrix} \right]+\left[ \begin{matrix} u_{k}^{\mathrm{sr}} \\ u_{k}^{\mathrm{cr}} \end{matrix} \right]+\left[ \begin{matrix} e_{k}^{\mathrm{sr}} \\ e_{k}^{\mathrm{cr}} \end{matrix} \right]$$

$$\mathrm{Var}\left[ \begin{matrix} u_{k}^{\mathrm{sr}} \\ u_{k}^{\mathrm{cr}} \end{matrix} \right]=\left[ \begin{matrix} \tau_{\mathrm{sr}}^{2} & \rho\tau_{\mathrm{sr}}\tau_{\mathrm{cr}} \\ \rho\tau_{\mathrm{sr}}\tau_{\mathrm{cr}} & \tau_{\mathrm{cr}}^{2} \end{matrix} \right].$$

Where $\mu^{\mathrm{sr}}$ and $\mu^{\mathrm{cr}}$ are the overall effects for self-reports and clinician ratings, respectively.

Second, in trials reporting more than one instrument per type of rating (clinician/self-report), we selected one instrument per study, giving priority to the most frequently used across studies. For this analysis, effects were again pooled using the bivariate correlated random-effects model described above. Additionally, to test the robustness of our primary analysis, we performed a third sensitivity analysis by using lower levels of assumed correlations in the variance-covariance matrices of the main model (*ρ*=0.6 among self-reports and *ρ*=0.5 between self-reports and clinician ratings).

The results of these sensitivity analyses are reported in Table S2 (Supplement).

## eResults

### Figure S1. PRISMA Flowchart

Date of searches: 01-01-2023


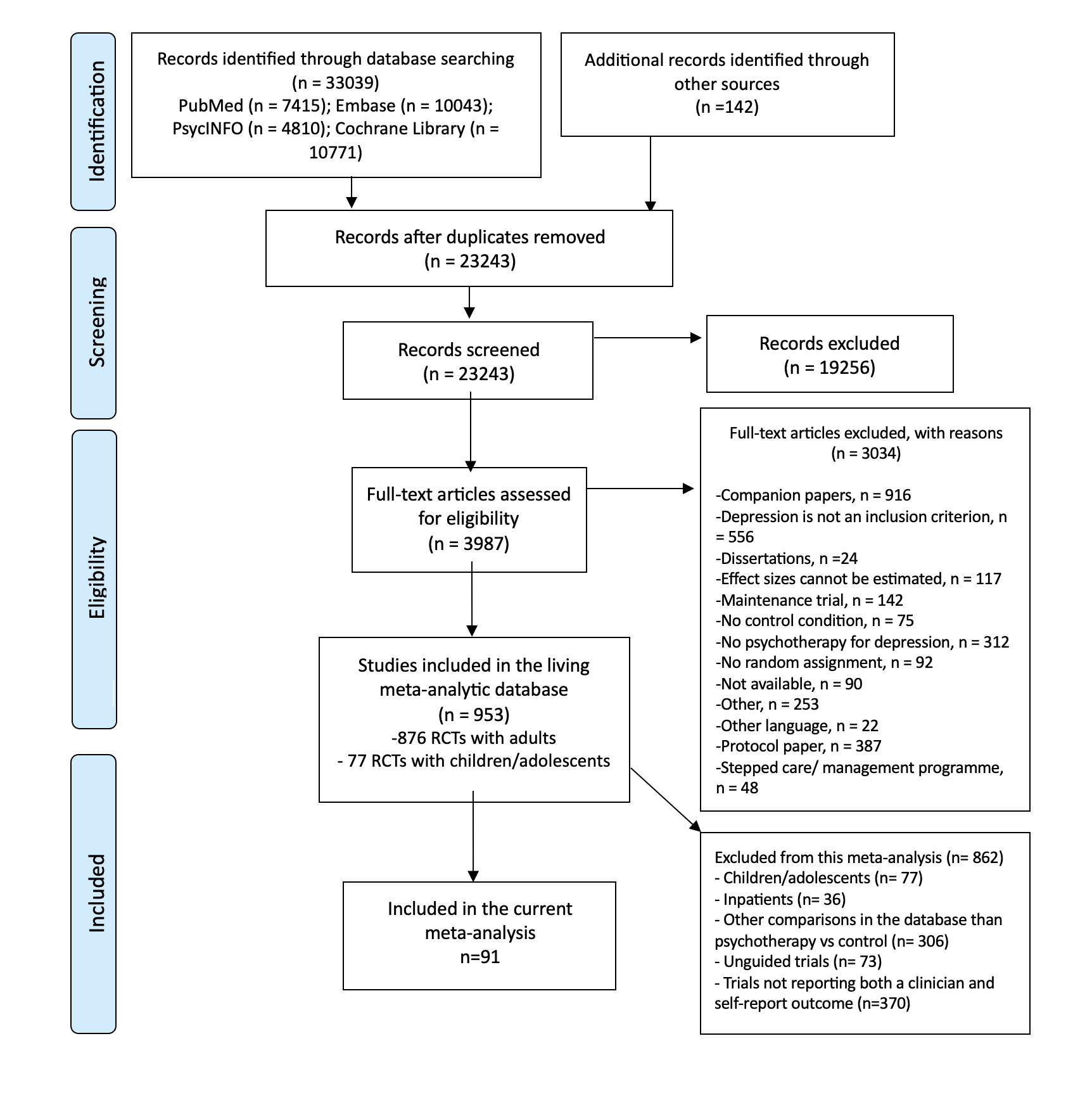


### References of the included studies

| Ammerman RT, Putnam FW, Altaye M, Stevens J, Teeters AR, Van Ginkel JB. A clinical trial of in-home CBT for depressed mothers in home visitation. Behavior Therapy. 2013;44(3):359-72. |
| --- |
| Arean PA, Perri MG, Nezu AM, Schein RL, Christopher F, Joseph TX. Comparative effectiveness of social problem-solving therapy and reminiscence therapy as treatments for depression in older adults. Journal of Consulting and Clinical Psychology. 1993;61(6):1003-10. |
| Ayen I, Hautzinger M. Cognitive behavior therapy for depression in menopausal women. A controlled, randomized treatment study. Zeitschrift fur Klinische Psychologie und Psychotherapie. 2004;33(4):290-9. |
| Baumeister, H., et al. (2021). "Effectiveness of a Guided Internet- and Mobile-Based Intervention for Patients with Chronic Back Pain and Depression (WARD-BP): A Multicenter, Pragmatic Randomized Controlled Trial." Psychother Psychosom 90(4): 255-268. |
| Bowman D, Scogin F, Lyrene B. The efficacy of self-examination therapy and cognitive bibliotherapy in the treatment of mild to moderate depression. Psychotherapy Research. 1995;5(2):131-40. |
| Carr, A., Finnegan, L., Griffin, E., Cotter, P., & Hyland, A. (2017). A Randomized Controlled Trial of the Say Yes to Life (SYTL) Positive Psychology Group Psychotherapy Program for Depression: An Interim Report. Journal of Contemporary Psychotherapy, 47(3), 153-161. |
| Castonguay LG, Schut AJ, Aikens DE, Constantino MJ, Laurenceau J-P, Bologh L, et al. Integrative cognitive therapy for depression: A preliminary investigation. Journal of Psychotherapy Integration. 2004;14(1):4-20. |
| Chan AS, Wong QY, Sze SL, Kwong PP, Han YM, Cheung MC. A Chinese Chan-based mind-body intervention for patients with depression. Journal of Affective Disorders. 2012;142(1-3):283-9. |
| Chiang KJ, Chen TH, Hsieh HT, Tsai JC, Ou KL, Chou KR. One-year follow-up of the effectiveness of cognitive behavioral group therapy for patients' depression: A randomized, single-blinded, controlled study. Scientific World Journal. 2015;2015:Article ID 373149. |
| Cohen S, O'Leary KD, Foran H. A randomized clinical trial of a brief, problem-focused couple therapy for depression. Behavior Therapy. 2010;41(4):433-46. |
| Desautels C, Savard J, Ivers H, Savard MH, Caplette-Gingras A. Treatment of depressive symptoms in patients with breast cancer: a randomized controlled trial comparing cognitive therapy and bright light therapy. Health psychology 2018; 37(1): 1‐13. |
| Dimidjian S, Hollon SD, Dobson KS, Schmaling KB, Kohlenberg RJ, Addis ME, et al. Randomized trial of behavioral activation, cognitive therapy, and antidepressant medication in the acute treatment of adults with major depression. Journal of Consulting and Clinical Psychology. 2006;74(4):658-70. |
| Dindo L, Recober A, Marchman JN, Turvey C, O'Hara MW. One-day behavioral treatment for patients with comorbid depression and migraine: A pilot study. Behaviour Research and Therapy. 2012;50(9):537-43. |
| Dobkin RD, Menza M, Allen LA, Gara MA, Mark MH, Tiu J, et al. Cognitive-behavioral therapy for depression in Parkinson's disease: A randomized, controlled trial. American Journal of Psychiatry. 2011;168(10):1066-74. |
| Dobkin RD, Mann SL, Gara MA, Interian A, Rodriguez KM, Menza M. Telephone-based cognitive behavioral therapy for depression in Parkinson disease: A randomized controlled trial. Neurology. 2020;94(16):e1764-e1773. |
| Dobkin, R. D., et al. (2021). "Innovating Parkinson's Care: A Randomized Controlled Trial of Telemedicine Depression Treatment." Movement Disorders 36(11): 2549-2558. |
| Dong, X., Sun, G., Zhan, J., Liu, F., Ma, S., Li, P., . . . Liu, Y. (2019). Telephone-based reminiscence therapy for colorectal cancer patients undergoing postoperative chemotherapy complicated with depression: a three-arm randomised controlled trial. Supportive care in cancer : official journal of the Multinational Association of Supportive Care in Cancer, 27(8), 2761-2769. doi:10.1007/s00520-018-4566-6 |
| Ebert DD, Buntrock C, Lehr D, et al. Effectiveness of Web- and Mobile-Based Treatment of Subthreshold Depression With Adherence-Focused Guidance: a Single-Blind Randomized Controlled Trial. Behavior therapy 2018; 49(1): 71‐83. |
| Elkin I, Shea MT, Watkins JT, Imber SD, Sotsky SM, Collins JF, et al. National institute of mental health treatment of depression collaborative research program. General effectiveness of treatments. Archives of General Psychiatry. 1989;46(11):971-82; discussion 83. |
| Fann JR, Bombardier CH, Vannoy S, Dyer J, Ludman E, Dikmen S, et al. Telephone and in-person cognitive behavioral therapy for major depression after traumatic brain injury: A randomized controlled trial. Journal of neurotrauma. 2015;32(1):45-57. |
| Floyd M, Scogin F, McKendree-Smith NL, Floyd DL, Rokke PD. Cognitive therapy for depression: A comparison of individual psychotherapy and bibliotherapy for depressed older adults. Behavior modification. 2004;28(2):297-318. |
| Fonagy P, Rost F, Carlyle JA, McPherson S, Thomas R, Pasco Fearon RM, et al. Pragmatic randomized controlled trial of long-term psychoanalytic psychotherapy for treatment-resistant depression: The Tavistock Adult Depression Study (TADS). World Psychiatry. 2015;14(3):312-21. |
| Forand NR, Barnett JG, Strunk DR, Hindiyeh MU, Feinberg JE, Keefe JR. Efficacy of Guided iCBT for Depression and Mediation of Change by Cognitive Skill Acquisition. Behavior Therapy 2018; 49(2): 295-307. |
| Freedland KE, Skala JA, Carney RM, Rubin EH, Lustman PJ, D·vila-Rom·n VG, et al. Treatment of depression after coronary artery bypass surgery: A randomized controlled trial. Archives of General Psychiatry. 2009;66(4):387-96. |
| Freedland KE, Carney RM, Rich MW, Steinmeyer BC, Rubin EH. Cognitive behavior therapy for depression and self-care in heart failure patients: A randomized clinical trial. JAMA Internal Medicine. 2015;175(11):1773-82. |
| Gellis ZD, McGinty J, Tierney L, Jordan C, Burton J, Misener E. Randomized controlled trial of problem-solving therapy for minor depression in home care. Research on Social Work Practice. 2008;18(6):596-606. |
| Gellis ZD, Bruce ML. Problem solving therapy for subthreshold depression in home healthcare patients with cardiovascular disease. The American Journal of Geriatric Psychiatry. 2010;18(6):464-74. |
| Gibbons MB, Thompson SM, Scott K, Schauble LA, Mooney T, Thompson D, et al. Supportive-expressive dynamic psychotherapy in the community mental health system: A pilot effectiveness trial for the treatment of depression. Psychotherapy (Chicago, Ill). 2012;49(3):303-16. |
| Greenberg J, Datta T, Shapero BG, Sevinc G, Mischoulon D, Lazar SW. Compassionate hearts protect against wandering minds: Self-compassion moderates the effect of mind-wandering on depression. Spirituality in Clinical Practice 2018; 5(3): 155-69. |
| Han YMY, Sze SL, Wong QY, Chan AS. A mind-body lifestyle intervention enhances emotional control in patients with major depressive disorder: a randomized, controlled study. Cognitive, affective & behavioral neuroscience. 2020;20(5):1056-1069. |
| Harley R, Sprich S, Safren S, Jacobo M, Fava M. Adaptation of dialectical behavior therapy skills training group for treatment-resistant depression. Journal of Nervous and Mental Disease. 2008;196(2):136-43. |
| Hautzinger M, Welz S. Kognitive Verhaltenstherapie bei Depressionen im Alter: Ergebnisse einer kontrollierten Vergleichsstudie unter ambulanten Bedingungen an Depressionen mittleren Schweregrads. = Cognitive behavioral therapy for depressed older outpatients: A controlled, randomized trial. Zeitschrift für Gerontologie und Geriatrie. 2004;37(6):427-35. |
| Hummel, J., Weisbrod, C., Boesch, L., Himpler, K., Hauer, K., Hautzinger, M., . . . Kopf, D. (2017). AIDE–Acute Illness and Depression in Elderly Patients. Cognitive Behavioral Group Psychotherapy in Geriatric Patients With Comorbid Depression: A Randomized, Controlled Trial. Journal of the american medical directors association, 18(4), 341-349. |
| Husain, N., Zulqernain, F., Carter, L.-A., Chaudhry, I., Fatima, B., Kiran, T., . . . Rahman, A. (2017). Treatment of maternal depression in urban slums of Karachi, Pakistan: a randomized controlled trial (RCT) of an integrated maternal psychological and early child development intervention. Asian journal of psychiatry, 29, 63-70. |
| Husain, N., et al. (2021). "Efficacy of learning through play plus intervention to reduce maternal depression in women with malnourished children: A randomized controlled trial from Pakistan(✰)." J Affect Disord 278: 78-84. |
| Jarrett RB, Schaffer M, McIntire D, Witt-Browder A, Kraft D, Risser RC. Treatment of atypical depression with cognitive therapy or phenelzine: A double-blind, placebo-controlled trial. Archives of General Psychiatry. 1999;56(5):431-7. |
| Johnson, J. E., Stout, R. L., Miller, T. R., Zlotnick, C., Cerbo, L. A., Andrade, J. T., . . . Wiltsey-Stirman, S. (2019). Randomized cost-effectiveness trial of group interpersonal psychotherapy (IPT) for prisoners with major depression. Journal of consulting and clinical psychology, 87(4), 392-406. doi:10.1037/ccp0000379 |
| Kanter JW, Santiago-Rivera AL, Santos MM, Nagy G, López M, Hurtado GD, et al. A randomized hybrid efficacy and effectiveness trial of behavioral activation for latinos with depression. Behavior Therapy. 2015;46(2):177-92. |
| Laidlaw K, Davidson K, Toner H, Jackson G, Clark S, Law J, et al. A randomised controlled trial of cognitive behaviour therapy vs treatment as usual in the treatment of mild to moderate late life depression. International Journal of Geriatric Psychiatry. 2008;23(8):843-50. |
| Larcombe NA, Wilson PH. An evaluation of cognitive-behaviour therapy for depression in patients with multiple sclerosis. The British Journal of Psychiatry. 1984;145:366-71. |
| Lee, E., et al. (2021). "Community-Based Multi-Site Randomized Controlled Trial of Behavioral Activation for Patients with Depressive Disorders." Community mental health journal. |
| Liu, H. and Y. Yang (2021). "Effects of a psychological nursing intervention on prevention of anxiety and depression in the postpartum period: a randomized controlled trial." Annals of General Psychiatry 20(1). |
| Lynch, T. R., Hempel, R. J., Whalley, B., Byford, S., Chamba, R., Clarke, P., . . . Russell, I. T. (2019). Refractory depression - mechanisms and efficacy of radically open dialectical behaviour therapy (RefraMED): findings of a randomised trial on benefits and harms. The British journal of psychiatry : the journal of mental science, 1-9. doi:10.1192/bjp.2019.53 |
| Matsuzaka, C., Wainberg, M., Norcini, P. A., Hoffmann, E., Coimbra, B., Braga, R., . . . Mello, M. (2017). Task shifting interpersonal counseling for depression: a pragmatic randomized controlled trial in primary care. BMC Psychiatry, 17(1) |
| McIndoo CC, File AA, Preddy T, Clark CG, Hopko DR. Mindfulness-based therapy and behavioral activation: A randomized controlled trial with depressed college students. Behaviour Research and Therapy. 2016;77:118-28. |
| Michalak J, Schultze M, Heidenreich T, Schramm E. A randomized controlled trial on the efficacy of mindfulness-based cognitive therapy and a group version of cognitive behavioral analysis system of psychotherapy for chronically depressed patients. Journal of Consulting and Clinical Psychology. 2015;83(5):951-63. |
| Mohr DC, Carmody T, Erickson L, Jin L, Leader J. Telephone-administered cognitive behavioral therapy for veterans served by community-based outpatient clinics. Journal of Consulting and Clinical Psychology. 2011;79(2):261-5. |
| Moon, J. R., et al. (2021). "The effects of rational emotive behavior therapy for depressive symptoms in adults with congenital heart disease." Heart Lung 50(6): 906-913. |
| Mulcahy R, Reay RE, Wilkinson RB, Owen C. A randomised control trial for the effectiveness of group interpersonal psychotherapy for postnatal depression. Archives of Women's Mental Health. 2010;13(2):125-39. |
| Mynors-Wallis L, Gath D, Lloyd-Thomas A, Tomlinson D. Randomised controlled trial comparing problem solving treatment with amitriptyline and placebo for major depression in primary care. BMJ. 1995;310(6977):441-5. |
| Nakagawa, A., Mitsuda, D., Sado, M., Abe, T., Fujisawa, D., Kikuchi, T., . . . Ono, Y. (2017). Effectiveness of supplementary cognitive-behavioral therapy for pharmacotherapy-resistant depression: A randomized controlled trial. Journal of clinical psychiatry, 78(8), 1126-1135 |
| Nezu AM, Perri MG. Social problem-solving therapy for unipolar depression: An initial dismantling investigation. Journal of Consulting and Clinical Psychology. 1989;57(3):408-13. |
| Niedermoser DW, Kalak N, Kiyhankhadiv A, et al. Workplace-Related Interpersonal Group Psychotherapy to Improve Life at Work in Individuals With Major Depressive Disorders: a Randomized Interventional Pilot Study. Frontiers in psychiatry. 2020;11. |
| O'Hara MW, Stuart S, Gorman LL, Wenzel A. Efficacy of interpersonal psychotherapy for postpartum depression. Archives of General Psychiatry. 2000;57(11):1039-45. |
| Pecheur DR, Edwards KJ. A comparison of secular and religious versions of cognitive therapy with depressed Christian college students. Journal of Psychology and Theology. 1984. |
| Poleshuck EL, Gamble SA, Bellenger K, Lu N, Tu X, Sorensen S, et al. Randomized controlled trial of interpersonal psychotherapy versus enhanced treatment as usual for women with co-occurring depression and pelvic pain. Journal of psychosomatic research. 2014;77(4):264-72. |
| Prendergast J, Austin MP. Early childhood nurse-delivered cognitive behavioural counselling for post-natal depression. Australasian Psychiatry. 2001;9(3):255-9. |
| Propst LR, Ostrom R, Watkins P, Dean T, Mashburn D. Comparative efficacy of religious and nonreligious cognitive-behavioral therapy for the treatment of clinical depression in religious individuals. Journal of Consulting and Clinical Psychology. 1992;60(1):94-103. |
| Rehm LP, Kornblith SJ, O'Hara MW, Lamparski DM, Romano JM, Volkin JI. An evaluation of major components in a self-control therapy program for depression. Behavior modification. 1981;5(4):459-89. |
| Ritvo, P., et al. (2021). "Online Mindfulness-Based Cognitive Behavioral Therapy Intervention for Youth With Major Depressive Disorders: Randomized Controlled Trial." J Med Internet Res 23(3): e24380. |
| Rohan KJ, Roecklein KA, Lindsey KT, Johnson LG, Lippy RD, Lacy TJ, et al. A randomized controlled trial of cognitive-behavioral therapy, light therapy, and their combination for seasonal affective disorder. Journal of Consulting and Clinical Psychology. 2007;75(3):489-500. |
| Ross M, Scott M. An evaluation of the effectiveness of individual and group cognitive therapy in the treatment of depressed patients in an inner city health centre. The Journal of the Royal College of General Practitioners. 1985;35(274):239-42. |
| Rosso, I., Killgore, W., Olson, E., Webb, C., Fukunaga, R., Auerbach, R., . . . Rauch, S. (2017). Internet-based cognitive behavior therapy for major depressive disorder: a randomized controlled trial. Depress Anxiety, 34(3), 236-245. |
| Russell, A., Gaunt, D. M., Cooper, K., Barton, S., Horwood, J., Kessler, D., . . . et al. (2019). The feasibility of low-intensity psychological therapy for depression co-occurring with autism in adults: the Autism Depression Trial (ADEPT) â€“ a pilot randomised controlled trial. Autism. doi:10.1177/1362361319889272 |
| Safren SA, O'Cleirigh C, Tan JY, Raminani SR, Reilly LC, Otto MW, et al. A randomized controlled trial of cognitive behavioral therapy for adherence and depression (CBT-AD) in HIV-infected individuals. Health Psychology. 2009;28(1):1-10. |
| Safren SA, Bedoya CA, O'Cleirigh C, Biello KB, Pinkston MM, Stein MD, et al. Cognitive behavioural therapy for adherence and depression in patients with HIV: A three-arm randomised controlled trial. The Lancet HIV. 2016;3(11):e529-e38. |
| Safren, S. A., et al. (2021). "Treating depression and improving adherence in HIV care with task-shared cognitive behavioural therapy in Khayelitsha, South Africa: a randomized controlled trial." J Int AIDS Soc 24(10): e25823. |
| Savard J, Simard S, Giguere I, Ivers H, Morin CM, Maunsell E, et al. Randomized clinical trial on cognitive therapy for depression in women with metastatic breast cancer: Psychological and immunological effects. Palliative and Supportive Care. 2006;4(3):219-37. |
| Schramm E, Mack S, Thiel N, Jenkner C, Elsaesser M, Fangmeier T. Interpersonal psychotherapy vs treatment as usual for major depression related to work stress: A pilot randomized controlled study. Frontiers in Psychiatry. 2020;11. |
| Scogin F, Hamblin D, Beutler L. Bibliotherapy for depressed older adults: A self-help alternative. The Gerontologist. 1987;27(3):383-7. |
| Scogin F, Jamison C, Gochneaur K. Comparative efficacy of cognitive and behavioral bibliotherapy for mildly and moderately depressed older adults. Journal of Consulting and Clinical Psychology. 1989;57(3):403-7. |
| Scott C, Tacchi MJ, Jones R, Scott J. Acute and one-year outcome of a randomised controlled trial of brief cognitive therapy for major depressive disorder in primary care. The British Journal of Psychiatry. 1997;171:131-4. |
| Segre LS, Brock RL, O'Hara MW. Depression treatment for impoverished mothers by point-of-care providers: A randomized controlled trial. Journal of Consulting and Clinical Psychology. 2015;83(2):314-24. |
| Selmi PM, Klein MH, Greist JH, Sorrell SP, Erdman HP. Computer-administered cognitive-behavioral therapy for depression. American Journal of Psychiatry. 1990;147(1):51-6. |
| Sheeber, L., Feil, E., Seeley, J., Leve, C., Gau, J., Davis, B., . . . Allan, S. (2017). Mom-net: evaluation of an internet-facilitated cognitive behavioral intervention for low-income depressed mothers. Journal of Consulting and Clinical Psychology, 85(4), 355-366. |
| Simoni JM, Wiebe JS, Sauceda JA, Huh D, Sanchez G, Longoria V, et al. A preliminary RCT of CBT-AD for adherence and depression among HIV-positive Latinos on the U.S.-Mexico border: The Nuevo Dia study. AIDS and behavior. 2013;17(8):2816-29. |
| Spinelli MG, Endicott J. Controlled clinical trial of interpersonal psychotherapy versus parenting education program for depressed pregnant women. American Journal of Psychiatry. 2003;160(3):555-62. |
| Swartz HA, Frank E, Zuckoff A, Cyranowski JM, Houck PR, Cheng Y, et al. Brief interpersonal psychotherapy for depressed mothers whose children are receiving psychiatric treatment. American Journal of Psychiatry. 2008;165(9):1155-62. |
| Taylor CB, Conrad A, Wilhelm FH, Strachowski D, Khaylis A, Neri E, et al. Does improving mood in depressed patients alter factors that may affect cardiovascular disease risk? Journal of Psychiatric Research. 2009;43(16):1246-52. |
| Tovote KA, Fleer J, Snippe E, Peeters A, Emmelkamp PMG, Sanderman R, et al. Individual mindfulness-based cognitive therapy and cognitive behavior therapy for treating depressive symptoms in patients with diabetes: Results of a randomized controlled trial. Diabetes care. 2014;37(9):2427-34. |
| Town, J., Abbass, A., Stride, C., & Bernier, D. (2017). A randomised controlled trial of Intensive Short-Term Dynamic Psychotherapy for treatment resistant depression: the Halifax Depression Study. Journal of Affective Disorders, 214, 15-25. |
| van Schaik A, van Marwijk H, Adèr H, van Dyck R, de Haan M, Penninx B, et al. Interpersonal psychotherapy for elderly patients in primary care. The American Journal of Geriatric Psychiatry. 2006;14(9):777-86. |
| Verduyn C, Barrowclough C, Roberts J, Tarrier N, Harrington R. Maternal depression and child behaviour problems: Randomised placebo-controlled trial of a cognitive-behavioural group intervention. British Journal of Psychiatry. 2003;183(OCT.):342-8. |
| Watkins ER, Taylor RS, Byng R, Baeyens C, Read R, Pearson K, et al. Guided self-help concreteness training as an intervention for major depression in primary care: A Phase II randomized controlled trial. Psychological Medicine. 2012;42(7):1359-71. |
| Watt LM, Cappeliez P. Integrative and instrumental reminiscence therapies for depression in older adults: Intervention strategies and treatment effectiveness. Aging and Mental Health. 2000;4(2):166-77. |
| Wilson PH, Goldin JC, Charbonneau-Powis M. Comparative efficacy of behavioral and cognitive treatments of depression. Cognitive Therapy and Research. 1983;7(2):111-24. |
| Wright JH, Wright AS, Albano AM, Basco MR, Goldsmith LJ, Raffield T, et al. Computer-assisted cognitive therapy for depression: Maintaining efficacy while reducing therapist time. American Journal of Psychiatry. 2005;162(6):1158-64. |
| Zu S, Xiang Y-T, Liu J, Zhang L, Wang G, Ma X, et al. A comparison of cognitive-behavioral therapy, antidepressants, their combination and standard treatment for Chinese patients with moderate–severe major depressive disorders. Journal of Affective Disorders. 2014;152-154:262-7. |
| Berman, M. I., Park, J., Kragenbrink, M. E., & Hegel, M. T. (2022). Accept Yourself! A Pilot Randomized Controlled Trial of a Self-Acceptance-Based Treatment for Large-Bodied Women With Depression. Behav Ther, 53(5), 913-926. doi:10.1016/j.beth.2022.03.002 |
| Euteneuer, F., Neuert, M., Salzmann, S., Fischer, S., Ehlert, U., & Rief, W. (2022). Does psychological treatment of major depression reduce cardiac risk biomarkers? An exploratory randomized controlled trial. Psychological medicine, 1-15. doi:10.1017/S0033291722000447 |
| Shan, Q., Xinxin, S., Zhijuan, X., Rongjing, D., & Minjie, Z. (2022). Effects of Cognitive Behavior Therapy on Depression, Illness Perception, and Quality of Life in Atrial Fibrillation Patients. Frontiers in Psychiatry, 13. doi:10.3389/fpsyt.2022.830363 |

### Characteristics of the included studies

After examining a total of 23,243 references (after duplicates removal) and 3,987 full-text papers, a total of 953 trials were included in the larger meta-analytical database, from which 91 met criteria for the current study.

The 91 RCTs, which had 128 comparisons between psychotherapy and control conditions (due to trials with multiple arms), included a total of 7250 participants (4104 in treatment and 3146 in control groups). The most relevant characteristics of each study are presented in Table S1. Most participants were middle-aged adults (n=65, 71%) with a diagnosis of depression or mood disorder (n=58, 64%) recruited from the community (n=41, 45%). The most frequent type of psychological treatment was CBT (n=71, 55%), delivered individually (n=61, 47%), and compared to waitlist control (n=36, 40%) and care-as-usual (n= 45, 49%).

A total of 38 trials (42%) achieved a low risk of bias score in domain 1 (bias arising from the randomization process) and 58 (64%) trials were rated at low risk of bias in domain 2 (bias arising from deviations from the intended interventions). An appropriate handling of missing data (domain 3) was reported in 37 trials (41%). Only 14 trials (15%) were rated at low risk for selective outcome reporting (domain 5), due to most of them being not registered (*n*=51; 56%) or retrospectively registered (n=21; 23%). In total, only 7 (8%) trials had a low risk of bias score in all four RoB domains.

Most of the trials (n=74; 81%) specified that the personnel administering the clinician-rated scales at post-treatment were masked to treatment allocation. The remaining 17 trials (19%) were considered in our analyses as not masked, either not reporting information about masking (n=13) or explicitly stating that assessors were not masked (n=4).

### Table S1. Characteristics of the 91 included studies

| Study | Psy | Contr | Self-reports | Clinician-rated | Blind | Form | N sess | Country | M. age | % wom | Recr | Diag | Pop | D1 | D2 | D3 | D5 | RoB |
| --- | --- | --- | --- | --- | --- | --- | --- | --- | --- | --- | --- | --- | --- | --- | --- | --- | --- | --- |
| Ammerman, 2013 | cbt | cau | bdi-2, epds | hdrs-17 | + | ind | 11 | us | 21.9 | 1.00 | oth | mdd | ppd | l | l | l | s | s |
| Arean, 1993 | pst | wl | bdi-1, gds | hdrs-17 | + | grp | 12 | us | 66.5 | 0.75 | com | mdd | old | s | s | h | s | h |
| Arean, 1993 | lrt | wl | bdi-1, gds | hdrs-17 | + | grp | 12 | us | 66.5 | 0.75 | com | mdd | old | s | s | h | s | h |
| Ayen, 2004 | cbt | wl | bdi-1 | ids | + | grp | 12 | eu | 51.3 | 1.00 | com | mood | oth | s | l | l | s | s |
| Ayen, 2004 | sup | wl | bdi-1 | ids | + | grp | 12 | eu | 51.3 | 1.00 | com | mood | oth | s | l | l | s | s |
| Baumeister, 2021 | cbt | cau | phq-9 | hdrs-17, qids-cr | + | gsh | 6 | eu | 49.9 | 0.60 | oth | mdd | med | l | l | s | l | s |
| Berman, 2022 | 3rd | other ctr | phq-9 | hrds-17 | + | grp | 7 | us | 51 | 1.00 | com | mdd | oth | s | l | l | l | s |
| Bowman, 1995 | pst | wl | bdi-1 | hdrs-21 | NI | gsh | 4 | us | 36.2 | 0.63 | com | cut | adul | s | h | l | s | h |
| Bowman, 1995 | cbt | wl | bdi-1 | hdrs-21 | NI | gsh | 4 | us | 36.2 | 0.63 | com | cut | adul | s | h | l | s | h |
| Carr, 2017 | cbt | cau | bdi-2 | hdrs-17, madrs-cr | + | grp | 20 | eu | 41 | 0.66 | clin | mdd | adul | s | l | h | s | h |
| Castonguay, 2004 | cbt | wl | bdi-1 | hdrs-24 | + | ind | 16 | us | 38.8 | 0.75 | com | mdd | adul | s | l | l | s | s |
| Chan, 2012 | cbt | wl | bdi-2 | hdrs-17 | + | grp | 10 | eas | 46.4 | 0.82 | clin | mdd | adul | l | h | h | s | h |
| Chan, 2012 | other psy | wl | bdi-2 | hdrs-17 | + | grp | 10 | eas | 46.4 | 0.82 | clin | mdd | adul | l | h | h | s | h |
| Chiang, 2015 | cbt | cau | bdi-2 | hdrs-21 | + | grp | 12 | eas | 46.1 | 0.63 | clin | mood | adul | s | h | h | s | h |
| Cohen, 2010 | other psy | wl | bdi-2 | hdrs-24 | + | cpl | 5 | us | 43.2 | 1.00 | com | mood | oth | s | h | l | s | h |
| Desautels, 2017 | cbt | wl | bdi-2, hads-d | hdrs-17 | + | ind | 8 | can | 57.1 | 1.00 | oth | cut | med | l | l | l | s | s |
| Dimidjian, 2006 | bat | other ctr | bdi-1 | hdrs-17 | + | ind | 24 | us | 39.9 | 0.66 | com | mdd | adul | s | l | l | s | s |
| Dimidjian, 2006 | cbt | other ctr | bdi-1 | hdrs-17 | + | ind | 24 | us | 39.9 | 0.66 | com | mdd | adul | s | l | l | s | s |
| Dindo, 2012 | 3rd | wl | idas-d | hdrs-17 | + | grp | 1 | us | 32.8 | 0.93 | oth | mdd | med | h | l | l | s | h |
| Dobkin, 2011 | cbt | cau | bdi-1 | hdrs-17 | + | ind | 10 | us | 64.6 | 0.40 | com | mood | med | l | l | l | s | s |
| Dobkin, 2020 | cbt | cau | bdi-1 | hdrs-17 | + | tel | 10 | us | 65.6 | 0.54 | oth | mood | med | l | l | l | h | h |
| Dobkin, 2021 | cbt | cau | bdi-2 | hdrs-17 | + | tel | 10 | us | 66.8 | 0.00 | com | mdd | med | l | l | l | l | l |
| Dong, 2019 | lrt | cau | sds | hdrs-24 | + | tel | 6 | eas | 59.1 | 0.50 | oth | cut | med | l | h | l | s | h |
| Ebert, 2018 | other psy | wl | ces-d | hdrs-24, qids-cr-16 | + | gsh | 5 | eu | 44.2 | 0.80 | com | sub | adul | l | l | s | h | h |
| Elkin, 1989 | ipt | other ctr | bdi-1 | hdrs-17 | + | ind | 13 | us | 35 | 0.70 | clin | mdd | adul | s | h | h | s | h |
| Elkin, 1989 | cbt | other ctr | bdi-1 | hdrs-17 | + | ind | 13 | us | 35 | 0.70 | clin | mdd | adul | s | h | h | s | h |
| Euteneuer, 2022 | cbt | wl | bdi-2 | madrs-cr | + | ind | 14 | eu | 30.3 | 0.60 | com | mdd | adul | s | l | s | s | h |
| Fann, 2015 | cbt | cau | scl-20 | hdrs-17 | + | tel | 10 | us | 45.8 | 0.37 | com | mdd | med | s | l | l | s | s |
| Fann, 2015 | cbt | cau | scl-20 | hdrs-17 | + | ind | 9 | us | 45.8 | 0.37 | com | mdd | med | s | l | l | s | s |
| Floyd, 2004 | cbt | wl | gds | hdrs-21 | + | ind | 16 | us | 68 | 0.76 | com | mood | old | s | h | h | s | h |
| Floyd, 2004 | cbt | wl | gds | hdrs-21 | + | gsh | 4 | us | 68 | 0.76 | com | mood | old | s | h | h | s | h |
| Fonagy, 2015 | dyn | cau | bdi-2 | hdrs-17 | + | ind | 60 | uk | 44.3 | 0.66 | clin | chr | adul | l | l | l | s | s |
| Forand, 2018 | cbt | wl | phq-9 | hdrs-17 | - | gsh | 6 | us | 33. | 0.75 | com | cut | adul | s | l | s | l | s |
| Freedland, 2009 | cbt | cau | bdi-1 | hdrs-17 | + | ind | 11 | us | 60.6 | 0.50 | oth | mood | med | l | l | l | s | s |
| Freedland, 2009 | sup | cau | bdi-1 | hdrs-17 | + | ind | 8 | us | 60.6 | 0.50 | oth | mood | med | l | l | l | s | s |
| Freedland, 2015 | cbt | cau | bdi-2, promis | hdrs-17 | + | ind | 11 | us | 55.8 | 0.46 | oth | mdd | med | l | l | l | s | s |
| Gellis, 2008 | pst | cau | gds | hdrs-17 | + | ind | 6 | us | 77.4 | 0.87 | oth | sub | med | s | s | s | s | h |
| Gellis, 2010 | pst | cau | bdi-1 | hdrs-17 | + | ind | 6 | us | 75.9 | 0.92 | oth | sub | med | l | l | l | s | s |
| Gibbons, 2012 | dyn | cau | basis-24-d | hdrs-17 | - | ind | 7 | us | 41.2 | 0.87 | clin | cut | adul | s | h | h | s | h |
| Greenberg, 2018 | 3rd | wl | bdi-2 | hdrs-28 | + | grp | 7 | us | 38.5 | 0.63 | com | cut | adul | s | h | h | h | h |
| Han, 2020 | cbt | wl | bdi-2 | hdrs-17 | + | grp | 10 | eas | 46.9 | 0.77 | clin | mdd | adul | l | h | h | s | h |
| Han, 2020 | other psy | wl | bdi-2 | hdrs-17 | + | grp | 10 | eas | 46.9 | 0.77 | clin | mdd | adul | l | h | h | s | h |
| Harley, 2008 | 3rd | wl | bdi-1 | hdrs-17 | + | grp | 14 | us | 41.8 | 0.75 | clin | chr | adul | s | h | h | s | h |
| Hautzinger, 2004 | cbt | wl | gds, scl-90-d | ids | + | grp | 12 | eu | 68.53 | 0.79 | com | mood | old | s | h | s | s | h |
| Hummel, 2017 | cbt | wl | hads-d | hdrs-17 | + | grp | 13 | eu | 81.94 | 0.80 | oth | cut | med | s | l | s | s | s |
| Husain, 2017 | cbt | cau | epds | hdrs-17 | + | grp | 6 | oth | 27.73 | 1.00 | oth | mood | ppd | l | l | l | l | l |
| Husain, 2021b | cbt | cau | epds | hdrs-17 | NI | grp | 10.0 | oth | 27.00 | 1.00 | oth | mood | ppd | s | h | s | s | h |
| Jarrett, 1999 | cbt | other ctr | bdi-1 | hdrs-21 | + | ind | 20.0 | us | 39.60 | 0.68 | com | mdd | adul | l | l | h | s | h |
| Johnson, 2019 | ipt | cau | qids-sr | hdrs-17 | + | oth | 24.0 | us | 39.00 | 0.35 | oth | mdd | oth | l | l | l | s | s |
| Kanter, 2015 | bat | cau | bdi-2 | hdrs-17 | + | ind | 8.0 | us | 38.10 | 0.79 | clin | mdd | oth | s | l | h | s | h |
| Laidlaw, 2008 | cbt | cau | bdi-2, gds | hdrs-17 | + | ind | 8.0 | uk | 74.03 | 0.73 | clin | mdd | old | l | h | h | s | h |
| Larcombe, 1984 | cbt | wl | bdi-1 | hdrs-17 | NI | grp | 6.0 | au | 42.50 | 0.68 | com | mood | med | s | h | h | s | h |
| Lee, 2021 | bat | cau | ces-d | hdrs-17 | + | grp | 10.0 | eas | 37.00 | 0.58 | clin | mood | adul | l | l | l | s | s |
| Liu, 2021 | cbt | cau | epds | hdrs | NI | ind | 6.0 | eas | 27.00 | 1.00 | oth | sub | ppd | l | h | s | h | h |
| Lynch, 2019 | other psy | cau | phq-9 | hdrs-17 | + | oth | 43.0 | uk | nr | 0.66 | clin | chr | adul | s | l | l | s | s |
| Matsuzaka, 2017 | ipt | cau | phq-9 | hdrs-17 | + | ind | 4.0 | oth | 43.84 | 0.94 | clin | mood | adul | h | l | s | h | h |
| McIndoo, 2016 | bat | wl | bdi-2 | hdrs-17 | NI | ind | 4.0 | us | 19.20 | 0.62 | com | cut | stud | l | l | l | s | s |
| McIndoo, 2016 | 3rd | wl | bdi-2 | hdrs-17 | NI | ind | 4.0 | us | 19.20 | 0.62 | com | cut | stud | l | l | l | s | s |
| Michalak, 2015 | other psy | cau | bdi-1 | hdrs-24 | + | oth | 10.0 | eu | 50.84 | 0.62 | com | chr | adul | l | l | l | l | l |
| Michalak, 2015 | 3rd | cau | bdi-1 | hdrs-24 | + | grp | 8.0 | eu | 50.84 | 0.62 | com | chr | adul | l | l | l | l | l |
| Mohr, 2011 | cbt | cau | phq-9 | hdrs-17 | + | tel | 16.0 | us | 55.90 | 0.09 | clin | mdd | oth | s | l | l | s | s |
| Moon, 2021 | cbt | cau | bdi-1 | hdrs-17 | + | grp | 8.0 | eas | 32.00 | 0.52 | oth | cut | med | l | l | l | s | s |
| Mulcahy, 2010 | ipt | cau | bdi-2, epds | hdrs-17 | + | oth | 11.0 | au | 32.22 | 1.00 | clin | mdd | ppd | s | h | h | s | h |
| Mynors-Wallis, 1995 | pst | other ctr | bdi-1 | hdrs | + | ind | 6.0 | uk | 37.10 | 0.77 | clin | mdd | adul | l | h | h | s | h |
| Nakagawa, 2017 | cbt | cau | bdi-2, qids-sr | grid-HDRS17 | + | ind | 15.0 | eas | 40.60 | 0.36 | clin | chr | adul | l | l | l | l | l |
| Nezu, 1989 | pst | wl | bdi-1 | hdrs-17 | + | grp | 10.0 | us | 41.73 | 0.77 | com | mdd | adul | s | h | h | s | h |
| Niedermoser, 2020 | ipt | cau | bdi-2 | hdrs-24 | NI | grp | 8.0 | eu | 40.86 | 50.00 | com | mdd | oth | l | l | h | s | h |
| O'Hara, 2000 | ipt | wl | bdi-1 | hdrs-21 | - | ind | 12.0 | us | 29.55 | 1.00 | oth | mdd | ppd | s | l | s | s | h |
| Pecheur, 1984 | cbt | wl | bdi-1 | hdrs-17 | + | ind | 8.0 | us | 24.00 | 0.90 | com | mdd | stud | s | h | h | s | h |
| Poleshuck, 2014 | ipt | cau | bdi-1 | hdrs-17 | NI | ind | 4.0 | us | 36.70 | 1.00 | oth | mdd | med | l | l | l | s | s |
| Prendergast, 2001 | cbt | other ctr | epds | madrs-cr | NI | ind | 6.0 | au | 32.20 | 1.00 | oth | mood | ppd | s | l | l | s | s |
| Propst, 1992 | cbt | wl | bdi-1 | hdrs-17 | + | ind | 19.0 | us | 40.00 | 0.83 | com | cut | oth | s | h | h | s | h |
| Propst, 1992 | cbt | cau | bdi-1 | hdrs-17 | + | ind | 19.0 | us | 40.00 | 0.83 | com | cut | oth | s | h | h | s | h |
| Rehm, 1981 | other psy | wl | bdi-1, mmpi-d | hdrs-17 | + | grp | 7.0 | us | 39.20 | 1.00 | com | cut | adul | s | h | h | s | h |
| Ritvo, 2021 | 3rd | cau | bdi-2, qids-sr | hdrs-24 | + | gsh | nr | can | 24.49 | 0.62 | clin | mdd | yadul | l | l | l | l | l |
| Rohan, 2007 | cbt | wl | bdi-2 | hdrs-21 | + | oth | 10.0 | us | 45.00 | 0.90 | com | mdd | adul | s | l | s | s | h |
| Rohan, 2007 | cbt | wl | bdi-2 | hdrs-21 | + | grp | 10.0 | us | 45.00 | 0.90 | com | mdd | adul | s | l | s | s | h |
| Ross, 1985 | cbt | wl | bdi-1 | madrs-cr | + | oth | 12.0 | uk | 33.00 | 0.63 | clin | mdd | adul | s | l | h | s | h |
| Rosso, 2017 | cbt | other ctr | phq-9 | hdrs-17 | + | gsh | 6.0 | us | 29.00 | 0.69 | com | mdd | adul | l | l | l | l | l |
| Russell, 2020 | bat | cau | bdi-2, phq-9 | grid-HDRS17 | + | gsh | 8.0 | uk | 37.71 | 0.27 | oth | cut | oth | l | h | h | l | h |
| Safren, 2009 | cbt | wl | bdi-1 | hdrs-17 | + | ind | 10.0 | us | nr | 0.16 | com | mood | med | s | l | s | s | h |
| Safren, 2016 | cbt | cau | ces-d | madrs-cr | + | ind | 11.0 | us | 47.45 | 0.31 | com | mood | med | s | l | l | l | s |
| Safren, 2016 | sup | cau | ces-d | madrs-cr | + | ind | 11.0 | us | 47.45 | 0.31 | com | mood | med | s | l | l | l | s |
| Safren, 2021 | cbt | cau | ces-d | hdrs-17 | + | ind | 8.0 | oth | nr | 0.70 | oth | mdd | med | s | l | l | l | s |
| Savard, 2006 | cbt | wl | bdi-1, hads-d | hdrs-17 | + | ind | 8.0 | can | 51.55 | 1.00 | com | cut | med | l | l | s | s | s |
| Schramm, 2020 | ipt | cau | bdi-2 | hdrs-24 | + | grp | 8.0 | eu | 47.40 | 0.79 | clin | mdd | oth | s | l | h | l | h |
| Scogin, 1987 | cbt | wl | bdi-1, gds | hdrs-21 | NI | gsh | 4.0 | us | 70.54 | 0.79 | com | cut | old | s | h | h | s | h |
| Scogin, 1987 | cbt | other ctr | bdi-1, gds | hdrs-21 | NI | gsh | 4.0 | us | 70.54 | 0.79 | com | cut | old | s | h | h | s | h |
| Scogin, 1989 | cbt | wl | gds | hdrs-17 | NI | gsh | 4.0 | us | 68.34 | 0.85 | com | cut | old | s | h | s | s | h |
| Scott, 1997 | cbt | cau | bdi-1 | hdrs-17 | + | ind | 6.0 | uk | 41.00 | 0.67 | clin | mdd | adul | s | h | h | s | h |
| Segre, 2015 | other psy | wl | epds, idas-gd | hdrs-17 | + | ind | 5.0 | us | 26.30 | 1.00 | oth | cut | oth | s | l | s | s | h |
| Selmi, 1990 | cbt | wl | bdi-1, scl-90-d | hdrs-17 | + | gsh | 6.0 | us | 28.20 | 0.64 | com | mood | adul | s | l | l | s | s |
| Selmi, 1990 | cbt | wl | bdi-1, scl-90-d | hdrs-17 | + | ind | 6.0 | us | 28.20 | 0.64 | com | mood | adul | s | l | l | s | s |
| Shan, 2022 | cbt | cau | phq-9 | hrds-17 | + | ind | 10.0 | eas | 62.81 | 0.64 | oth | mood | med | l | h | s | s | h |
| Sheeber, 2017 | cbt | other ctr | phq-9 | hdrs-17 | + | gsh | 7.0 | us | 31.80 | 1.00 | com | cut | ppd | l | l | l | l | l |
| Simoni, 2013 | cbt | cau | bdi-1 | madrs-cr | + | ind | 11.0 | us | 46.00 | 0.28 | com | cut | med | l | l | l | s | s |
| Spinelli, 2003 | ipt | other ctr | bdi-1, epds | hdrs-17 | NI | ind | 16.0 | us | 28.80 | 1.00 | com | mdd | ppd | s | l | h | s | h |
| Swartz, 2008 | ipt | cau | bdi-1 | hdrs-17 | NI | ind | 9.0 | us | 42.7 | 1.00 | com | mdd | oth | s | h | h | s | h |
| Taylor, 2009 | cbt | wl | bdi-1 | hdrs | + | ind | 15.0 | us | 62.2 | 0.67 | com | cut | med | s | l | h | s | h |
| Tovote, 2014 | 3rd | wl | bdi-2 | hdrs-7 | - | ind | 8 | eu | 53.1 | 0.49 | oth | cut | med | s | l | h | s | h |
| Tovote, 2014 | cbt | wl | bdi-2 | hdrs-7 | - | ind | 8 | eu | 53.1 | 0.49 | oth | cut | med | s | l | h | s | h |
| Town, 2017 | dyn | cau | phq-9 | hdrs-17 | + | ind | 16 | can | 41.5 | 0.63 | clin | chr | adul | l | l | l | s | s |
| van Schaik, 2006 | ipt | cau | gds | madrs-cr | + | ind | 8 | eu | 67.9 | 0.69 | clin | mdd | old | l | l | s | s | s |
| Verduyn, 2003 | cbt | cau | bdi-1 | hdrs-17 | + | grp | 16 | uk | 29.8 | 1.00 | oth | cut | oth | l | l | h | s | h |
| Verduyn, 2003 | sup | cau | bdi-1 | hdrs-17 | + | grp | 16 | uk | 29.8 | 1.00 | oth | cut | oth | l | l | h | s | h |
| Watkins, 2012 | other psy | cau | bdi-2, phq-9 | hdrs-17 | + | oth | 7 | uk | 43.6 | 0.64 | clin | mood | adul | l | l | s | s | s |
| Watkins, 2012 | other psy | other ctr | bdi-2, phq-9 | hdrs-17 | + | oth | 7 | uk | 43.6 | 0.64 | clin | mood | adul | l | l | s | s | s |
| Watt, 2000 | lrt | other ctr | gds | hdrs-17 | + | grp | 6 | can | 68.6 | 0.54 | com | cut | old | s | h | h | s | h |
| Wilson, 1983 | bat | wl | bdi-1 | hdrs-17 | NI | ind | 8 | au | 39.5 | 0.80 | com | cut | adul | s | h | h | s | h |
| Wilson, 1983 | cbt | wl | bdi-1 | hdrs-17 | NI | ind | 8 | au | 39.5 | 0.80 | com | cut | adul | s | h | h | s | h |
| Wright, 2005 | cbt | wl | bdi-1 | hdrs-17 | + | gsh | 9 | us | 40.2 | 0.76 | com | mdd | adul | s | l | h | s | h |
| Wright, 2005 | cbt | wl | bdi-1 | hdrs-17 | + | ind | 9 | us | 40.2 | 0.76 | com | mdd | adul | s | l | h | s | h |
| Zu, 2014 | cbt | cau | qids-sr | hdrs-17 | + | ind | 20 | eas | 38.5 | 0.51 | clin | mdd | adul | s | h | h | s | h |

**Types of psychotherapy**: cbt: cognitive behavioural therapy, ipt: interpersonal psychotherapy; 3rd: third wave therapies; sup: supportive therapy; dyn: psychodynamic therapy; bat: behavioural activation therapy; pst: problem solving therapy; lrt: life review therapy; other psy: other psychotherapy. **Types of controls:** wl: waitlist, cau: care as usual, other ctr: other type of control (e.g., attention placebo). **Characteristics of the trials and participants**: Blind: if clinicians administering the assessment are blinded (“+” is yes, “-“ is no, and “NI” is no information); N sess: Number of sessions; % wom: Percentage of women in the trial sample; Recruit: Recruitment (com= community, clin= clinical, oth= other); Diagn: Diagnosis of depression at inclusion (cut= above a cut-off in a symptoms scale, mdd= major depressive disorder, mood = mood disorder, chr= chronic depression, sub= subclinical depression); Pop: Target population of the trial (adul: middle-aged adults, ppd: perinatal depression, med: individuals with comorbid medical conditions, stud: students, yadul: young adults, old: older adults, oth: other specific target groups).

**Risk of bias:** “h” denotes high risk of bias, “l” denotes low risk, and “s” denotes some concerns. D1: randomization process, D2: deviations from the intended interventions, D3: missing outcome data, D5: Selection of the reported result. For D4 (measurement of the outcome) we collected information regarding the blinding of clinicians (see column “Blind”) .

### List of instruments used in the included studies

| HRSD | Hamilton Rating Scale for Depression (Hamilton, 1960) |
| --- | --- |
| MADRS-cr | Montgomery-Åsberg Depression Rating Scale (Montgomery & Åsberg, 1979) |
| IDS | Inventory of Depressive Symptomatology (Rush et al., 1986) |
| QIDS-cr | Quick Inventory of Depressive Symptomatology (Rush et al., 2003) |
| BDI-I | Beck Depression Inventory (Beck, Ward, Mendelson, Mock, & Erbaugh, 1961) |
| BDI-II | Beck Depression Inventory-II; (Beck, Steer, & Brown, 1996) |
| GDS | Geriatric Depression Scale (Yesavage, 1988) |
| PHQ-9 | Patient Health Questionnaire (Kroenke, Spitzer, & Williams, 2001) |
| EPDS | Edinburgh Postnatal Depression Scale (Cox, Holden, & Sagovsky, 1987) |
| CES-D | Center for Epidemiologic Studies Depression Scale (Radloff, 1977) |
| SCL-90 | Symptom Checklist 90 (Derogatis, Lipman, & Covi, 1973) |
| QIDS-sr | Quick Inventory of Depressive Symptomatology (Rush et al., 2003) |
| MMPI-D | Minnesota Multiphasic Personality Inventory – Depression Scale (Hathaway & McKinley, 1951) |
| HADS-D | Hospital Anxiety and Depression Scale – Depression subscale (Zigmond & Snaith, 1983) |
| IDAS-D | Inventory of Depression and Anxiety Symptoms (Watson et al., 2007) |
| SDS | Zung Self-Rating Depression Scale (Zung, 1965) |
| PROMIS | NIH-Patient-Reported Outcomes Measurement Information System (Cella et al., 2007) |
| BASIS-24-D | Behavior and Symptom Identification Scale (Eisen, Normand, Belanger, Spiro III, & Esch, 2004) |

References of the instruments can be found in page 22.

### Table S2. Sensitivity analysis on methodological decisions

We conducted three **sensitivity analyses on methodological decisions**. Two of them involved using different methods for pooling outcomes: 1) pre-aggregating the instruments on the rating level (self-report vs. clinician), and 2) selecting one instrument per study (based on frequency). In the 3) third sensitivity analysis we repeated our main model for pooling (four-level hierarchical meta-analysis model) but assuming a different level of correlation between ratings for the variance-covariance matrices (*ρ*=0.6 among self-reports and ρ=0.5 between self-reports and clinician ratings).

| **Contrasts** | ***Δg*** | **95% CI** | **PI** | ***p_contrasts_*** |
| --- | --- | --- | --- | --- |
| 1) Pre-aggregating on rating level *(*Δ*g)* | 0,131 | 0,04 to 0,22 | -1,19 to 1,44 | 0,005 |
| 2) One instrument per study *(*Δ*g)* | 0,148 | 0,06 to 0,24 | -1,26 to 1,55 | 0,001 |
| 3) Different correlations *(*Δ*g)* | 0,113 | 0,03 to 0,20 | -1,08 to 1,30 | 0,009 |

Notes

*Δg =* Differential effects between self-reports and clinician-rated instruments. A positive value indicates larger effects for clinician-rated instruments.

95% CI: 95% Confidence Interval; PI: Prediction Interval

### Table S3. Heterogeneity in the models (*I*^2^ and tau^2^)

This table shows heterogeneity estimates (*I*^2^ and tau^2^) across different models and levels within the models. Our **main pooling method** (“main model”) is a four-level hierarchical meta-analysis model. For this model, we assumed a doubly nested random effects structure (effects *in* [clinician, self-report] outcomes *in* studies), which means that three heterogeneity variance components are estimated across these levels: study, rating (self-report vs. clinician), and outcome or instrument level (effect size for a specific instrument).

Next, we conducted three **sensitivity analyses on methodological decisions**. Two of them involved using different methods for pooling outcomes: 1) pre-aggregating the instruments on the rating level (self-report vs. clinician), and 2) selecting one instrument per study (based on frequency). This resulted in two heterogeneity variance components, based on the rating (self-report vs. clinician). The 3) third sensitivity analysis on a methodological decision involved the level of correlation assumed in the variance-covariance matrices of the main model (*ρ*=0.6 among self-reports and ρ=0.5 between self-reports and clinician ratings). The pooling in this analysis was the same as in our main model, thus resulting in three heterogeneity variance components.

We also conducted **sensitivity analyses based on important trial and participant characteristics**, namely 4) blinding of assessors, 5) specific population subgroups, and 6) excluding the GDS and EPDS (two self-report scales specifically focused on geriatric and perinatal depression, respectively). For these all analyses we used the same pooling method as in our main model, resulting in the same three levels of heterogeneity variance components.

| Model | Level | tau^2^ | *I*^2^ |
| --- | --- | --- | --- |
| Main model | Study | 0,241 | 59,28 |
|  | Rating | 0,016 | 3,90 |
|  | Instrument | 0,074 | 18,13 |
| *Sensitivity analyses on methodological decisions* | | | |
| 1) Pre-aggregating | Self-report | 0,264 | 84,94 |
|  | Clinician | 0,435 | 89,34 |
| 2) One instrument per study | Self-report | 0,270 | 81,22 |
|  | Clinician | 0,495 | 88,64 |
| 3) Different levels of correlations | Study | 0,279 | 65,33 |
|  | Rating | 0,000 | 7,10 |
|  | Instrument | 0,072 | 16,91 |
| *Sensitivity analyses based on trial and participant characteristics* | | | |
| 4) Blinding of assessors | Study | 0,231 | 58,40 |
|  | Rating | 0,015 | 3,87 |
|  | Instrument | 0,074 | 18,57 |
| 5) Specific population subgroups | Study | 0,286 | 65,88 |
|  | Rating | 0,000 | 0,000 |
|  | Instrument | 0,072 | 16,57 |
| 6) Excluding GDS and EPDS | Study | 0,195 | 57,51 |
|  | Rating | 0,000 | 0,000 |
|  | Instrument | 0,067 | 19,67 |
| 7) Only trials reporting BDI and HRSD | Study | 0,173 | 47,02 |
|  | Rating | 0,000 | 0,000 |
|  | Instrument | 0,09 | 25,16 |

### Multimodel inference: best models

We employed a meta-analytic multimodel inference technique (Anderson, 2007; Buckland et al., 1997) to explore if there were study characteristics that predicted the degree to which patient and clinician-rated outcomes differ in a study. In the first step of multimodel inference, a set of putative predictors is defined. In our analysis, we pre-specified the following potential moderators of the effect size difference between patient and clinician-reported outcomes: masking of the assessor (masked vs. unmasked, considering self-reports as unmasked), overall risk of bias score (high risk/some concerns vs. low risk), target group (specific subgroup vs general adults), control group (waitlist vs. other control groups), country (western vs non-western), and type of treatment (cbt vs other). The next step involved fitting a separate (meta-regression) model for each possible combination of these predictors. This means that the effect of each predictor is estimated not once, but in many models, including more complex multivariable models that control for the effect of other predictors. Based on the fit of each model (as measured by the corrected Akaike Information Criterion; AICc), it is then possible to create a weighted average for each variable, representing its importance in predicting effect size differences across all fitted models. Lower AICc means that the model fits better.

The following table presents the **best 10 models derived from the multimodel inference analysis,** with meta-regression models restricted to a maximum of 6 terms, leading to a total of 58700 fitted models.

| **Model** | **AICC** | **Weights** |
| --- | --- | --- |
| yi ~ 1 + waitlist + specific_subgroup + non_western + rating_clinician:specific_subgroup | 400.8632 | 0.08884671 |
| yi ~ 1 + waitlist + specific_subgroup + non_western + non_cbt + rating_clinician:specific_subgroup | 401.0533 | 0.08078898 |
| yi ~ 1 + blinded + waitlist + specific_subgroup + non_western + rating_clinician:specific_subgroup | 402.4824 | 0.03953949 |
| yi ~ 1 + waitlist + specific_subgroup + non_western + non_cbt + rating_clinician:specific_subgroup + rating_clinician:non_cbt | 402.5933 | 0.03740684 |
| yi ~ 1 + blinded + waitlist + specific_subgroup + non_western + non_cbt + rating_clinician:specific_subgroup | 402.6548 | 0.03627438 |
| yi ~ 1 + waitlist + specific_subgroup + non_western + rating_clinician:specific_subgroup + rating_clinician:non_western | 402.6752 | 0.03590613 |
| yi ~ 1 + waitlist + specific_subgroup + non_western + rating_clinician:waitlist + rating_clinician:specific_subgroup | 402.6846 | 0.03573812 |
| yi ~ 1 + high_rob + waitlist + specific_subgroup + non_western + rating_clinician:specific_subgroup | 402.7260 | 0.03500562 |
| yi ~ 1 + high_rob + waitlist + specific_subgroup + non_western + non_cbt + rating_clinician:specific_subgroup | 402.7548 | 0.03450472 |
| yi ~ 1 + waitlist + specific_subgroup + non_western + non_cbt + rating_clinician:specific_subgroup + rating_clinician:non_western | 402.8792 | 0.03242352 |

### References of the instruments

Beck, A. T., Ward, C. H., Mendelson, M., Mock, J., & Erbaugh, J. (1961). An inventory for measuring depression. *Archives of general psychiatry, 4*(6), 561-571.

Cella, D., Yount, S., Rothrock, N., Gershon, R., Cook, K., Reeve, B., . . . Rose, M. (2007). The Patient-Reported Outcomes Measurement Information System (PROMIS): progress of an NIH Roadmap cooperative group during its first two years. *Med Care, 45*(5 Suppl 1), S3-s11. doi:10.1097/01.mlr.0000258615.42478.55

Cox, J. L., Holden, J. M., & Sagovsky, R. (1987). Detection of postnatal depression. Development of the 10-item Edinburgh Postnatal Depression Scale. *Br J Psychiatry, 150*, 782-786. doi:10.1192/bjp.150.6.782

Derogatis, L. R., Lipman, R. S., & Covi, L. (1973). SCL-90: an outpatient psychiatric rating scale--preliminary report. *Psychopharmacology Bulletin, 9*(1), 13-28. Retrieved from https://www.scopus.com/inward/record.uri?eid=2-s2.0-0015541555&partnerID=40&md5=461d5ec0bbd52a13c3c0864963e91eb4

Eisen, S. V., Normand, S.-L., Belanger, A. J., Spiro III, A., & Esch, D. (2004). The revised behavior and symptom identification scale (BASIS-R): reliability and validity. *Medical care, 42*(12), 1230-1241.

Hamilton, M. (1960). A rating scale for depression. *Journal of neurology, neurosurgery, and psychiatry, 23*(1), 56.

Hathaway, S. R., & McKinley, J. C. (1951). Minnesota multiphasic personality inventory; manual, revised.

Montgomery, S. A., & Åsberg, M. (1979). A new depression scale designed to be sensitive to change. *The British journal of psychiatry, 134*(4), 382-389.

Radloff, L. S. (1977). The CES-D Scale:A Self-Report Depression Scale for Research in the General Population. *Applied psychological measurement, 1*(3), 385-401. doi:10.1177/014662167700100306

Rush, A. J., Giles, D. E., Schlesser, M. A., Fulton, C. L., Weissenburger, J., & Burns, C. (1986). The Inventory for Depressive Symptomatology (IDS): preliminary findings. *Psychiatry Res, 18*(1), 65-87. doi:10.1016/0165-1781(86)90060-0

Rush, A. J., Trivedi, M. H., Ibrahim, H. M., Carmody, T. J., Arnow, B., Klein, D. N., . . . Keller, M. B. (2003). The 16-Item Quick Inventory of Depressive Symptomatology (QIDS), clinician rating (QIDS-C), and self-report (QIDS-SR): a psychometric evaluation in patients with chronic major depression. *Biol Psychiatry, 54*(5), 573-583. doi:10.1016/s0006-3223(02)01866-8

Watson, D., O'Hara, M. W., Simms, L. J., Kotov, R., Chmielewski, M., McDade-Montez, E. A., . . . Stuart, S. (2007). Development and validation of the Inventory of Depression and Anxiety Symptoms (IDAS). *Psychol Assess, 19*(3), 253-268. doi:10.1037/1040-3590.19.3.253

Yesavage, J. A. (1988). Geriatric Depression Scale. *Psychopharmacol Bull, 24*(4), 709-711.

Zigmond, A. S., & Snaith, R. P. (1983). The Hospital Anxiety and Depression Scale. *Acta Psychiatrica Scandinavica, 67*(6), 361-370. doi:https://doi.org/10.1111/j.1600-0447.1983.tb09716.x

Zung, W. W. (1965). A self-rating depression scale. *Archives of general psychiatry, 12*(1), 63-70. doi:10.1001/archpsyc.1965.01720310065008

### GRADE assessments

The strength of evidence of our main findings was assessed following GRADE (Schünemann, Brożek, Guyatt & Oxman, 2013).

| **Analysis** | **k** | **n** | **SMD**  ***(Δg)*** | **95% CI** | **PI** | **RoB** | **Inconsistency** | **Imprecision** | **Indirectness** | **Publication bias** | **Overall** |
| --- | --- | --- | --- | --- | --- | --- | --- | --- | --- | --- | --- |
| Primary:  Self-reports vs Clinician-rated | 283 | 7250 | 0.12 | 0.03 to 0.21 | -1.04 to 1.27 | Serious | Serious | Not serious | Not serious | Not serious | ⨁⨁◯◯ Low |
| Self-reports vs. Unmasked clinicians | 49 | 1390 | 0.20 | -0.03 to 0.43 | -1.07 to 1.47 | Serious | Serious | Serious | Not serious | Not serious | ⨁◯◯◯ Very Low |
| Self-reports vs. Masked clinicians | 234 | 5749 | 0.10 | 0.00 to 0.20 | -1.04 to 1.24 | Serious | Serious | Not serious | Not serious | Not serious | ⨁⨁◯◯ Low |

Abbreviations: k= number of effect sizes; n= number of participants; SMD*(Δg)* = Standardized mean difference between self-reports and clinician-rated outcomes; 95% CI= 95% Confidence intervals; *I*^2^ (95% CI)= I-squared statistic measuring heterogeneity, with accompanying confidence intervals; PI = Prediction intervals; RoB= Risk of bias

- **RoB:** All analyses were downgraded one level in all analyses because only 7% of the included trials was rated at low RoB.
- **Inconsistency:** All analyses were downgraded one level due to substantial heterogeneity indicated by wide prediction intervals.
- **Imprecision:** We rated imprecision considering 1) the optimal information size, which was estimated to be a samlpe size of 400 participants to detect an effect size of 0.2 (with  α = 0.05 and β = 0.20), and 2) whether the 95% CI excludes no effect.
- **Indirectness:** We rated the generalizability of the current results to observer vs self ratings outside of the current study. We considered the included scales to be representative of the ones used commonly used in practice and clinical research.
- **Publication bias:** We did not downgrade for publication bias, as there is low risk for publication bias to affect our outcome of interest (difference between self-report and clinician ratings).

References

Schünemann H, Brożek J, Guyatt G, Oxman A (eds). GRADE Handbook; Handbook for grading the quality of evidence and the strength of recommendations using the GRADE approach. Updated October 2013. https://gdt.gradepro.org/app/handbook/handbook.html Accessed at January 7, 2025.
